# A DNA robotic switch with regulated autonomous display of cytotoxic ligand nanopatterns

**DOI:** 10.1038/s41565-024-01676-4

**Published:** 2024-07-01

**Authors:** Yang Wang, Igor Baars, Ieva Berzina, Iris Rocamonde-Lago, Boxuan Shen, Yunshi Yang, Marco Lolaico, Janine Waldvogel, Ioanna Smyrlaki, Keying Zhu, Robert A. Harris, Björn Högberg

**Affiliations:** 1 Department of Medical Biochemistry and Biophysics, Karolinska Institutet, Stockholm, Sweden; 2 Biohybrid Materials, Department of Bioproducts and Biosystems, Aalto University School of Chemical Engineering, Aalto, Finland; 3 Applied Immunology and Immunotherapy, Department of Clinical Neuroscience, Karolinska Institutet, Center for Molecular Medicine, Karolinska University Hospital, Stockholm, Sweden

**Keywords:** Nanofabrication and nanopatterning, Biosensors, Drug delivery

## Abstract

The clustering of death receptors (DRs) at the membrane leads to apoptosis. With the goal of treating tumours, multivalent molecular tools that initiate this mechanism have been developed. However, DRs are also ubiquitously expressed in healthy tissue. Here we present a stimuli-responsive robotic switch nanodevice that can autonomously and selectively turn on the display of cytotoxic ligand patterns in tumour microenvironments. We demonstrate a switchable DNA origami that normally hides six ligands but displays them as a hexagonal pattern 10 nm in diameter once under higher acidity. This can effectively cluster DRs and trigger apoptosis of human breast cancer cells at pH 6.5 while remaining inert at pH 7.4. When administered to mice bearing human breast cancer xenografts, this nanodevice decreased tumour growth by up to 70%. The data demonstrate the feasibility and opportunities for developing ligand pattern switches as a path for targeted treatment.

## Abstract (web summary)

Here the authors present a pH-sensitive DNA origami nanoswitch that hides ligands for death receptors and displays them as a cytotoxic hexagonal pattern in acidic tumour microenvironments. This reduces tumour growth in a murine model of breast cancer with minimal on-target, off-tumour toxicity.

## Main

Tumour-targeting strategies are plagued by the lack of ideal tumour-specific membrane receptors^1,2^. The tumour necrosis factor (TNF) receptor superfamily (TNFRSF) plays essential roles in mammalian physiology^3–5^. However, the ubiquitous expression of TNFRSF receptors on human cells prevents the development of biomolecules exclusively targeting specific cell populations via them. Nevertheless, ligands or antibodies of specific TNFRSF members have been developed to activate the apoptosis machinery of cancer cells^6,7^. Recent studies, however, revealed that these ligands or antibodies lack efficacy due to their inability to precisely tune the oligomerization of the receptors on which the apoptosis cascades rely^8–11^.

By conjugating ligands to different scaffolds, an effective way to oligomerize certain members of TNFRSF can be achieved^12–15^. Furthermore, to tune the inter-ligand distance with molecular precision, we and others have recently designed flat sheet-like DNA origamis for patterning ligands of death receptor 5 (DR5) or Fas receptors, revealing that hexagonal ligand patterns with specific inter-ligand distance at the nanoscale substantially drive apoptosis^16–18^. However, these ligand patterns also interact with healthy cells. It is thus expected that, when applied in vivo, they risk promoting on-target, off-tumour toxicities, including neurotoxicity^19^, increased susceptibility to infections^20^ and immune disorders^21^.

Cells of solid tumours exorbitantly consume oxygen and other nutrients, causing low oxygen tension and anaerobic glycolysis that ultimately leads to increased acidity^22^. In this Article, to address the potential side effects of TNFRSF ligand patterns when used in vivo, we have developed a pH-sensitive three-dimensional DNA origami switch that can sense the high acidity of solid tumours. The device autonomously switches on surface display of six ligands as a sub-10 nm hexagonal pattern, while at higher pH this pattern remains hidden inside a cavity.

## Design of the origami robotic switch

**Fig. 1:**
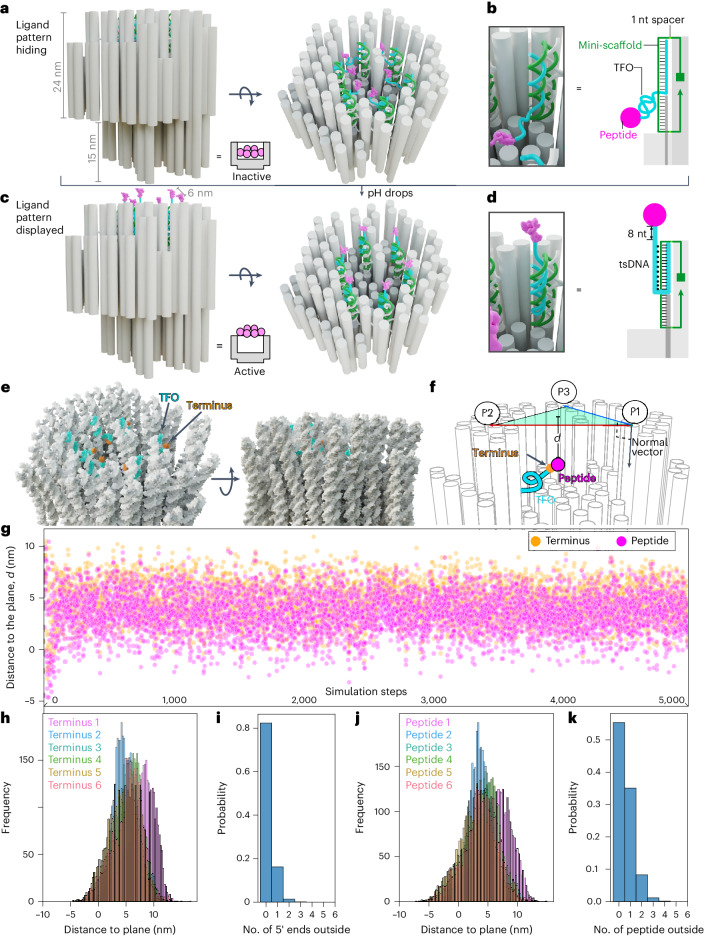
Design principles and simulations of the pH-responsive origami switch for ligand pattern hiding and display. **a**, A three-dimensional origami has a head part with a cavity that physically hides six peptide ligands conjugated to DNA strands. **b**, Illustration of the mechanism of how a peptide ligand hides inside the head cavity via the entropic spring imparted by the random coiling of the TFO. **c**, When the pH drops, the TFO of the peptide–DNA conjugate forms a tsDNA that forces the six peptides that were originally hidden in the cavity to get displayed as a hexagonal pattern on the top surface of the origami. **d**, Close-ups of the tsDNA. **e**, The mean structure of the head part and cavity (the whole mean structure is shown in Supplementary Fig. 2), calculated as an average from multiple oxDNA simulations. TFOs are coloured in cyan, and their 5′ termini are in orange. The top (left) and side (right) views are presented. **f**, Schematic of how the distance from the TFO terminus or the peptide to the origami surface plane is defined by two vectors. **g**, Real-time distances, from the TFO terminus (dots in orange) or from the peptide surface to the origami surface (dots in magenta, recalculated from the dataset of orange points via inclusion of the orientation information of the TFO terminus and the physical diameter of the peptide), along the simulation. **h**,**j**, Distance frequency histograms of the 5′ termini (**h**) or peptides (**j**) of the six TFOs to the origami surface. **i**,**k**, Probability that multiple 5′ termini (**i**) or peptides (**k**) of the six TFOs are simultaneously outside the origami. For **g**, **h** and **j**, positive values indicate that the tracked points are at the origami side of the defined plane (inside the origami cavity), while negative values mean that the tracked points are on the opposite side (outside the cavity). Source data

We designed the origami as an asymmetric double cylinder with a 24-nm-tall hollow head and a 15-nm-tall solid stem (Fig. 1a and Supplementary Fig. 1). The head has a 14-nm-deep cavity, where specific oligonucleotides (called ‘mini-scaffolds’ because they function as replacement for the scaffold in the origami design) are located to act as bridges between the typical origami staples and a binding site for ligand-decorated oligos. The distance between two adjacent mini-scaffolds is 6 nm. Each mini-scaffold is then used as a site to hybridize a peptide ligand-functionalized oligo, bearing one sequence that forms the double helix (the hybridization region) with the mini-scaffold as well as bearing an additional sequence of triplex-forming oligo (TFO).

At lower pH, the TFO can wind along the duplex to form a triple-stranded DNA (tsDNA) that occurs when purines pair simultaneously from both their Watson–Crick and Hoogsteen interfaces (Fig. 1b). Via controlling the AT content, the triggering pH of tsDNA can be finely tuned^23^. Because of its high programmability, Ng et al. used this mechanism to spatially organize plasmids into raster-filled structures^24^. In addition, Sachenbacher et al. elucidated the structural basis of triplex domains and duplex–triplex crossovers in origami^25^. As we can customize the mini-scaffolds, we can subsequently control the responsive pH of tsDNA.

Our design intent was to create a structure that could display an apoptosis-driving ligand pattern in solid tumours (pH 6.5) while physically hiding ligands under normal physiological conditions (pH 7.4) (Fig. 1c). Previous studies have utilized tsDNA to switch the overall configuration of origami^23,26–28^. In these cases, the formation of tsDNA under low pH brings two domains of origami closer while the high pH releases them. For example, Ijäs et al. prepared the reconfigurable DNA origami nanocapsule^26^. However, the cargo got displayed under high pH while hidden under low pH, which is the opposite of what would be needed for drug exposure in acidic conditions. Our design reverses this.

Moreover, to reduce potential steric hindrance from the origami and improve the accessibility of the exposed hexagonal ligand pattern to corresponding receptors on cells, we introduced eight extra bases between the ligand and the TFO (Fig. 1d).

## Ligand hiding estimation using molecular dynamics simulation

We performed molecular dynamics simulations using oxDNA^29,30^, to estimate if peptides can be hidden inside the origami cavity. Because of entropic spring effects, single-stranded DNA (ssDNA) tends to coil up rather than stay linearly stretched^31^. It indeed reveals that TFOs typically stay in coiled configurations, hidden in the origami (Fig. 1e and Supplementary Fig. 2).

To calculate the distances from the 5′ terminus of a TFO or from the conjugated peptide to the origami surface, during simulations, a plane on the origami surface is defined using two vectors, and the normal vector of the plane points towards the origami (Fig. 1f). Orientation of the TFO terminus and the van der Waal’s diameter (2.8 nm) of the peptide were introduced to calculate the relative distance between the peptide and the defined plane (Supplementary Fig. 3). The real-time distances indicated that, in most cases, both the 5′ termini of TFOs and the peptides remained inside the origami (Fig. 1g and Supplementary Fig. 4).

Each 5′ terminus can, with a low probability, transiently protrude outside the origami cavity (Fig. 1h), but the probability that more than three termini simultaneously end up at or above the surface is close to zero (Fig. 1i). The same holds true when also accounting for the added estimated position of the peptide (Fig. 1j,k). Our previous work with flat sheet-like origami indicated that, to be cytotoxic, more than three of the six hexagonally patterned peptides needed to be presented^18^. The very low probability for more than three peptides staying outside the origami thus predicts that, when the tsDNA does not form, our design can be assumed to not be cytotoxic, supporting our aim to use pH as a regulator for cytotoxicity.

## Origami characterization

Origami was stabilized using the ultraviolet (UV) cross-linking method^32,33^. We placed extra thymidines (Ts) at the termini and crossovers of staples, creating sites for cyclobutane pyrimidine dimer bonds. The ssDNA regions of the mini-scaffolds were designed to not contain any Ts and Cs. Samples, before and after cross-linking, under transmission electron microscopy (TEM) similarly showed the expected homogeneous particles (Supplementary Figs. 5–7). In 1× phosphate-buffered saline (PBS), the original origami showed some tendency to disassemble (Supplementary Fig. 8), while the cross-linked one kept its shape (Supplementary Figs. 9 and 10).

**Fig. 2:**
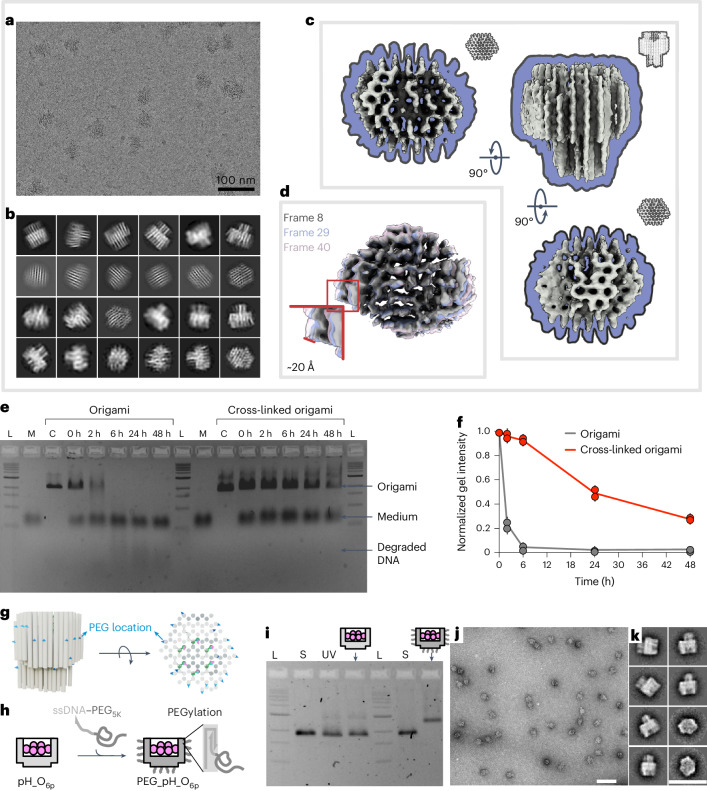
Structure characterization of the UV cross-linked origami and its stability. **a**, Example of cryo-EM micrograph of the UV cross-linked origami structure. Scale bar, 100 nm. **b**, Representative 2D class averages of the UV cross-linked origami structure. **c**, Cryo-EM density map. **d**, Frames picked from the 3DFlex analysis. **e**, A 2% agarose gel electrophoresis of origami incubated in freshly prepared DMEM with 10% FBS at 37 °C. L, 1 kb DNA ladder; M, DMEM with 10% FBS; C, origami before adding to the medium. **f**, Normalized agarose gel intensity versus time for origamis. Data are presented as mean ± s.d., *n* = 2 independent measurements. **g**, Protrusion sites (coloured in blue) surrounding the origami for site-specific PEGylation. **h**, The PEGylation mechanism, via hybridization of ssDNA–PEG_5K_ to 20 single-stranded protrusions of the origami. **i**, Electrophoresis of origamis in a 2% agarose gel. L, ladder; S, p8064 scaffold; UV, UV cross-linked origami, six-peptides-loaded device (pH_O_6p_) and PEGylated version (PEG_pH_O_6p_). **j**, Negative-stain TEM image of the PEG_pH_O_6p_. Scale bar, 100 nm. **k**, Two-dimensional class averages of the PEG_pH_O_6p_. Scale bar, 100 nm. Source data

The cryogenic electron microscopy (cryo-EM) data of the cross-linked origami yielded an intermediate-resolution reconstruction of 7 Å, sufficient to confirm the structural consistency between the experimentally observed and designed origami (Fig. 2a–c and Supplementary Fig. 11). The terminal regions of the individual helices could not be seen due to their extensive flexibility. However, the flexibility of the core part could be quantified and visualized using the 3DFlex tools in CryoSPARC^34,35^. This analysis showed the continuous flow of helices spanning across ~20 Å distance transversally (Fig. 2d), while no considerable flexing was observed longitudinally.

Stability tests in cell culture medium showed that around 30% of the cross-linked origami survived up to 48 h (Fig. 2e,f), indicating that the origami would be reliable for hours of cellular experiments.

We designed 20 ssDNA protrusions surrounding the origami for the docking of ssDNA–polyethylene glycol (PEG)_5K_ polymers (Fig. 2g,h). PEGylation is expected to improve the stability and circulation half-time of DNA origami when applied in vivo^36,37^. Our site-specific PEGylation here renders each origami a much lower PEG_5K_ coating density than the previous oligolysine-mediated PEGylation^37^. We hypothesized, though, that a site-specific PEGylation could help us avoid interference in TFO. The PEGylated UV-cross-linked origami migrated slower than the bare origami (Fig. 2i) on gel, as expected following successful PEGylation. TEM imaging (Fig. 2j) and averaged 2D classes (Fig. 2k) agreed with its morphology.

## pH-dependent tsDNA origami switch

We detected the ssDNA regions of mini-scaffolds using Cy5-tagged complementary oligo. It gave a stepwise pattern corresponding to the abundance of mini-scaffold per origami (Supplementary Fig. 12), indicating that mini-scaffolds were integrated.

**Fig. 3:**
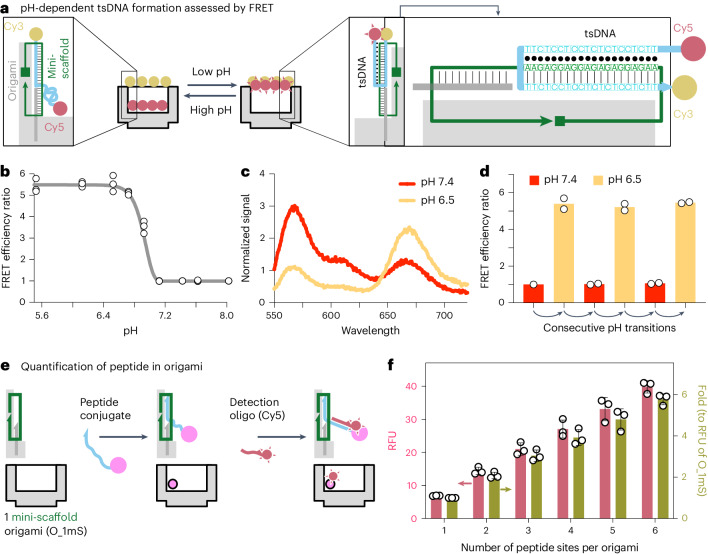
pH-sensitive FRET and peptide quantification. **a**, The principle of using FRET to verify the pH-dependent formation of the tsDNA inside the DNA origami. When a tsDNA forms, the donor chromophore (Cy3) and acceptor chromophore (Cy5) get in proximity, showing FRET. The sequence of the tsDNA is indicated to the right. **b**, Changes of normalized (to FRET efficiency under pH 7.4) FRET efficiency ratio in buffers with different pH (*n* = 3 independent measurements). **c**, With an excitation of 520 nm, the emission spectrum of the origami samples under pH 7.4 or 6.5. **d**, Normalized (to FRET efficiency under pH 7.4) FRET efficiency ratio when we consecutively exchanged the origami samples between buffer with pH 7.4 and 6.5 (*n* = 2 independent measurements). **e**, Illustration of how we quantify peptides in origami. After loading the ssDNA–peptide conjugate into the origami, the peptide was detected using Cy5-labelled oligo that targets the TFO of the conjugate. **f**, Peptide quantification of origami structures with different numbers of mini-scaffold with the principle indicated in **e** (*n* = 3 independent measurements). RFU, relative fluorescence units. Data are presented as mean ± s.d. Source data

We then introduced TFO to the mini-scaffold. Cy5 and Cy3 were tagged to the 5′ end and 3′ end, respectively, of the longer oligo that was hybridized to the mini-scaffold, allowing us to follow the switch-on and switch-off of the tsDNA via measuring Förster resonance energy transfer (FRET) (Fig. 3a). Its switch-on takes the 5′ end of the oligo to the surface of the origami, bringing the donor chromophore and acceptor chromophore in proximity. It showed that, when the AT content was 50%, the tsDNAs were mostly in the switch-on state at pH below 6.8 while they were in the switch-off state at pH 7.4 (Fig. 3b,c and Supplementary Fig. 13), demonstrating their responses to the extracellular acidity (pH 6.5–6.8) of solid tumours. When we changed the pH to 7.4 and 6.5, it showed a reversible change between the switch-off and switch-on states over several consecutive rounds (Fig. 3d).

## Quantification of ssDNA–ligand conjugates in origami

We conjugated the 5′ end of TFO with the TNF-related apoptosis-inducing ligand (TRAIL)-mimicking peptide ligand of DR5 (Supplementary Fig. 14). The affinity of the conjugate to DR5 stayed similar under pH 7.4 (*K*_D_ = 37.8 nM) or 6.5 (*K*_D_ = 52.8 nM), supported by surface plasmon resonance (SPR) measurements (Supplementary Fig. 15). Via hybridization, we assembled the conjugates into the cross-linked origami.

To verify the stoichiometry, we prepared origamis with different numbers of mini-scaffold and incubated them with the conjugate. We used a Cy5-oligo to target the TFO (Fig. 3e), showing that the fluorescence increased stepwise with the corresponding increase in mini-scaffolds (Supplementary Fig. 16). The analysis showed a clear correlation between the designed number of mini-scaffolds per origami and the fold change of the Cy5 signal (Fig. 3f), supporting that the peptide abundance per origami followed our design. Origami switch, containing six peptides, is referred to as pH_O_6p_. Its PEGylated version is referred to as PEG_pH_O_6p_.

## pH-driven ligand pattern induces DR5 clustering

**Fig. 4:**
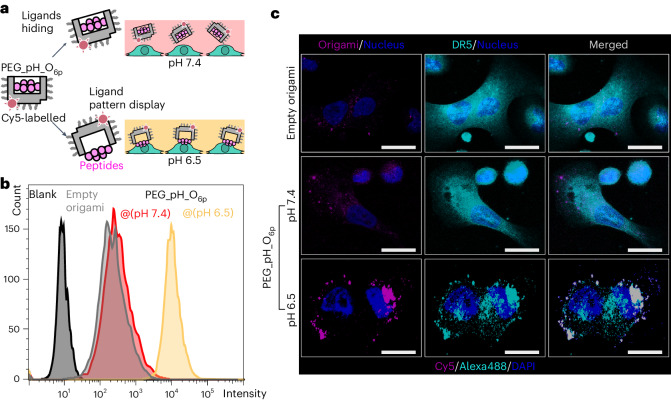
Interaction of the origami with breast cancer cells and induction of DR5 clustering. **a**, Experimental setup of cancer cell treatment with Cy5-labelled origami at pH 7.4 or 6.5. **b**, The Cy5 signal of SK-BR-3 cells treated with 5 nM empty origami, PEG_pH_O_6p_ at pH 7.4 or 6.5, measured by flow cytometry. **c**, Localization of Cy5-labelled origamis (magenta) relative to DR5 (cyan; detected using Alexa488-labelled anti-DR5 monoclonal antibody) on SK-BR-3 cells. The co-localization of the magenta and cyan is shown in grey. Maximum intensity projections from *z*-stacks. Scale bars, 20 μm.

The affinity (to DR5) of the origami always displaying the peptide pattern on surface was slightly higher than the case of non-structured peptides (Supplementary Fig. 17), which we hypothesize is positively, and negatively, contributed to by: (1) the patterning of peptides and (2) the large molecular mass of origami, respectively. We performed a pH-controlled cell experiment to verify PEG_pH_O_6p_ (Fig. 4a). SK-BR-3 cells were treated with Cy5-labelled PEG_pH_O_6p_ structures for 4 h and then analysed using flow cytometry. Cells at pH 6.5 showed a notably higher Cy5 signal than did cells at pH 7.4 (Fig. 4b and Supplementary Fig. 18). This confirmed that the peptides were presented outside the origami when the pH was low enough to form tsDNA, mediating origami structure to cells. Meanwhile, when it was under pH 7.4, cells incubated with empty origami had similarly low signals as those treated with PEG_pH_O_6p_, indicating the absence of DR5-mediated interactions and that peptides can be reliably hidden under pH 7.4.

We previously found that with the treatment of a sub-10 nm hexagonal pattern of peptide ligands, DR5 clustered into structurally diverse assemblies in the membranes of apoptotic cells^18^. We thus examined if the same effect was achieved with the origami robotic switch. This revealed that, with SK-BR-3 cells, treatment with PEG_pH_O_6p_ under pH 6.5 caused notably more clustering of DR5 than the treatment with empty origami or PEG_pH_O_6p_ under pH 7.4 (Fig. 4c). Under pH 6.5, the co-localization of signals from origami and DR5 clusters on cells supported that the receptor clustering was induced by the origami treatment. Furthermore, the morphology of cells treated with PEG_pH_O_6p_ under pH 6.5 showed shrinkage, which is one of the typical characteristics of apoptosis.

Based on a previous study^38^, we developed a repair qPCR protocol (Supplementary Fig. 19) to quantify origami associated with cells. It showed that, as expected, the total amount of PEG_pH_O_6p_ per cell was 16-fold higher under pH 6.5 versus both pH 7.4 and the case of empty origami. We also analysed intracellular origami with the same protocol following enzymatic DNA degradation of the extracellular material. This similarly showed a notable increase in internalization at low pH. Together, these assays demonstrated that the tumour microenvironment (TME)-corresponding acidity promoted the presentation of the ligand pattern from the origami, which in turn resulted in the binding to and clustering of DR5.

## Cytotoxicity of the origami switch is pH dependent

**Fig. 5:**
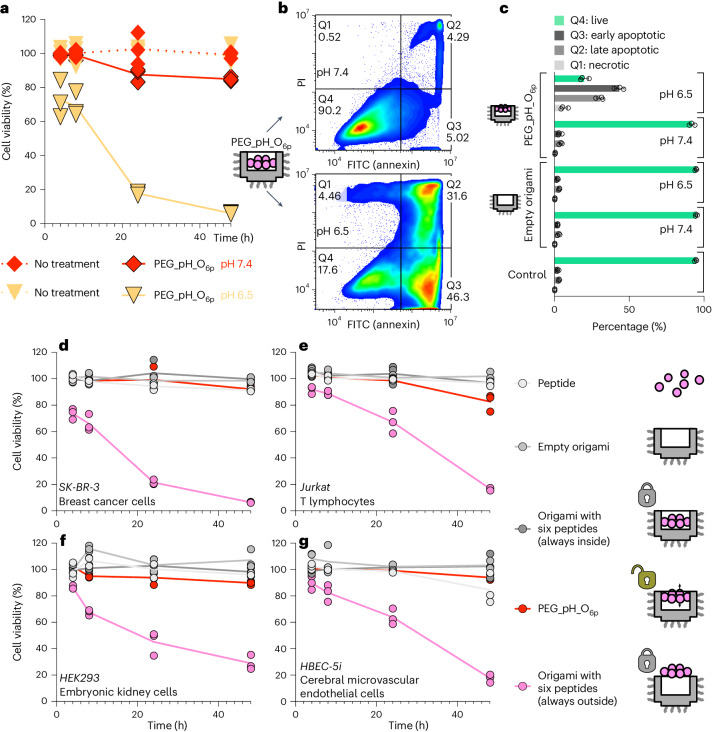
Cell cytotoxicity is controlled by acidic conditions. **a**, Cell viability assay on SK-BR-3 cancer cells after treatment with PEG_pH_O_6p_ origami at pH 7.4 or 6.5. **b**,**c**, Cancer cell apoptosis assay of DNA origami treatment at pH 7.4 or 6.5, measured by flow cytometry (Q1, necrotic cells; Q2, late apoptotic cells; Q3, early apoptotic cells; Q4, live cells). **d**–**g**, Cell viability assay at pH 7.4 of breast cancer cells (**d**) and human non-cancer cells, including T lymphocytes (**e**), kidney cells (**f**) and endothelial cells (**g**). *n* = 3 independent experiments. Data are presented as mean ± s.d. Source data

We first assessed if the pH itself could impact cell growth. There were no viability differences between the cells maintained under the original cell culture medium and medium at pH 7.4 or 6.5. SK-BR-3 cancer cells treated with PEG_pH_O_6p_ at pH 7.4 did not show an impact on cell viability. In contrast, cell viability at pH 6.5 continuously decreased after a 4 h incubation. After 24 and 48 h, less than 20% and 10% of cells survived, respectively (Fig. 5a). The further analysis on apoptosis demonstrated that substantially more cells incubated with PEG_pH_O_6p_ at pH 6.5 were in an early (46.8% versus 5.8%) or late (32.2% versus 4.5%) apoptotic state compared with cells incubated with PEG_pH_O_6p_ under pH 7.4, empty origami or control (Fig. 5b,c). We thus verified that the PEG_pH_O_6p_ switched on its apoptosis-inducing and cytotoxic bioactivity in a pH-dependent manner.

When considering in vivo treatment, the origami would interact with circulating immune cells or cells in healthy organs and tissues before accumulating in TME, and that DR5 is expressed in almost all human cells. If PEG_pH_O_6p_ cannot keep the cytotoxic ligand pattern hidden before reaching TME, it might cause unwanted side effects. At pH 7.4, apart from cancer cells (Fig. 5d), we tested the device for safety with human T lymphocytes (Fig. 5e), embryonic kidney cells (Fig. 5f) and cerebral microvascular endothelial cells (Fig. 5g). We also fabricated origami samples in which peptides were always hidden (negative control) or always displayed (positive control) as controls. Viability profiles of all these cells showed similar trends. Treatments using the negative control and treatment with PEG_pH_O_6p_ did not impact on cell viability, while cells in the positive control were decreasingly viable. This supports the notion that the designed peptide pattern can generally induce apoptosis-based cytotoxicity of human cells rather than being specific for cancer cells. Importantly, PEG_pH_O_6p_ showed similar effects as the negative control, demonstrating its biosafety before exposure to TME.

## Solid tumours get suppressed by PEG_pH_O_6p_

We tested the origami switch on nude mice implanted with SK-BR-3 xenografts. We administered the origami samples via intravenous (i.v.) or intratumoural (i.t.) injection.

**Fig. 6:**
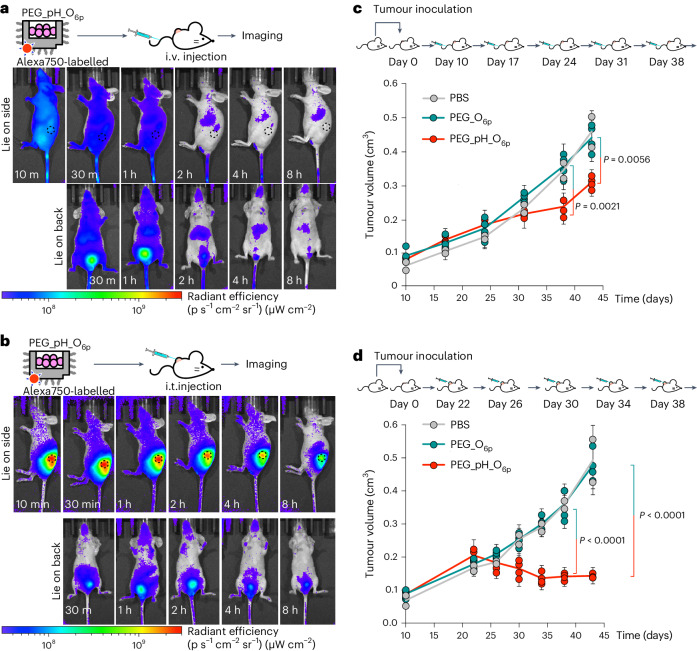
In vivo biodistribution and anti-tumour effects. **a**, Biodistribution imaging after i.v. injection with Alexa750-labelled PEG_pH_O_6p_ origami in nude mouse bearing a SK-BR-3 tumour at different timepoints. The tumour is indicated using a dashed circle. **b**, Imaging after i.t. injection with Alexa750-labelled PEG_pH_O_6p_ origami in nude mouse bearing a SK-BR-3 tumour at different timepoints. The tumour is indicated using a dashed circle. **c**, Treatment schedule and SK-BR-3 tumour growth curves of mice receiving i.v. injections of PBS (*n* = 2), PEG_mutated_O_6p_ (non-switchable control; *n* = 4) or PEG_pH_O_6p_ (*n* = 4). **d**, Treatment schedule and SK-BR-3 tumour growth curves of mice receiving i.t. injections of PBS (*n* = 2), PEG_mutated_O_6p_ (*n* = 4) or PEG_pH_O_6p_ (*n* = 4). Statistical analysis was performed with one-way ANOVA followed by Tukey post-tests. Data are presented as mean ± s.d. Source data

Biodistributions were assessed via tracking Alexa750-modified origami. Following the i.v. injection, the PEG_pH_O_6p_ spread throughout the whole body and displayed a rapid renal clearance (Fig. 6a). Its dominant signal accumulated in the bladder, where it reached a maximum after 1 h, and was excreted thereafter. Some liver accumulation was also noticeable. These results are similar to origami biodistribution experiments in a previous study using mice without tumours^37^. We observed no signs of selective accumulation in the solid tumour, which could be explained by the lack of tumour-targeting motifs in the origami. For the i.t. injection, origami signals mainly stayed in the tumour for more than 8 h, although they slowly decreased over time (Fig. 6b). Renal clearance was also evident, with the maximal signal in bladder after 2 h, but there was no strong accumulation in liver.

Finally, we treated mice with PEG_pH_O_6p_ to suppress the implanted tumours. Apart from the PBS group, PEG_mutated_O_6p_ was included as another control group. To get the PEG_mutated_O_6p_ control, the TFO sequence of PEG_pH_O_6p_ was mutated, making PEG_mutated_O_6p_ almost chemically identical to the switchable PEG_pH_O_6p_, but just the minute sequence mutations in the TFO region renders the control structure not sensitive to pH and, thus, always hiding peptides inside the origami.

Using i.v. administrations, the PEG_pH_O_6p_ started showing its effects after three injections, and it gradually slowed down the growth of tumour volumes by around 30% by the endpoint (Fig. 6c and Supplementary Fig. 20). For i.t. injections, the PEG_pH_O_6p_ suppressed solid tumours. Volumes of tumours receiving PEG_pH_O_6p_ substantially decreased; compared with the control groups, its tumour suppression efficacy was around 70% (Fig. 6d and Supplementary Fig. 21). With both routes of administration, tumours receiving PEG_mutated_O_6p_ treatments grew similarly to those in mice receiving PBS treatment, supporting that the anti-tumour mechanism of PEG_pH_O_6p_ relied on its pH sensitivity. Biomarkers of apoptosis, cleaved caspase-3 and cleaved caspase-8, of the tumours treated with PEG_pH_O_6p_ treatments were more abundant (Supplementary Fig. 22). Altogether, these treatment results demonstrated the apoptosis induction-based therapeutic effects of the DNA origami nanorobotic switch in solid tumours.

## Conclusions

We report a DNA origami switch that senses TME and then displays a cytotoxic ligand pattern. This method of autonomous actuation of cytotoxic ligand patterns could improve cancer treatment efficacy and reduce side effects, raising the possibility of developing new drugs. The addressability of DNA origami enables the precise patterning of biomolecules^39–41^. It has gained increasing interest for use in deciphering mechanisms of biology. However, limiting these patterns to specific conditions is difficult. Reconfigurable DNA nanostructures, which expose ligands upon opening, can help, but they face challenges in achieving high yields in both preparation and activation. Our pH-sensitive tsDNA-based origami switch overcomes these issues, showing substantial effectiveness in inducing apoptosis under acidic environments.

Our strategy aims to reduce on-target/off-tumour side effects, confirmed in vitro using non-cancer cells such as human T lymphocytes and others at pH 7.4. However, in vivo verification in mouse models is challenging due to species-specific peptide ligand binding. In our study, the peptide ligand did not affect non-tumour cells in BALB/c nude mice xenografts, but future studies should investigate this in humanized mouse models for a more accurate assessment.

DNA origami production costs are now reduced due to enzymatic and bacteriophage-based staple production, bringing the price to about $200 g^−1^ (refs. ^42,43^). Although our study used more expensive solid-phase synthesized staples for prototyping, using these cost-effective techniques can lower the cost for scale-up. Additionally, the recent availability of affordable peptide synthesis will further aid in scaling up DNA nanorobotics production for therapeutic tests in the future.

## Methods

### DNA origami preparation

#### DNA origami folding and purification

We used the phage-amplification-in-bacteria protocol to produce p8064 scaffold DNA^18^. Staple oligonucleotides were ordered from Integrated DNA Technologies (see ‘DNA oligo sequences of the origami.xlsx’ in Supplementary Data). A standard 100 μl folding mixture contained 20 nM p8064 scaffold DNA, 100 nM of each staple oligonucleotide, 22 mM MgCl_2_, 5 mM Tris and 1 mM EDTA. DNA origami formed during an annealing programme from 60 °C to 24 °C in 14 h. We used the PEG precipitation method described by previous papers^44^ to purify DNA origami. We resuspended the DNA origami pellet with the buffer containing 5 mM MgCl_2_, 5 mM NaCl, 5 mM Tris and 1 mM EDTA at 37 °C on a shaker, at 300 rpm, for 4 h.

#### DNA origami irradiation with UV

We adjusted concentrations of DNA origami to be at or below 100 nM. On an ice bath, we irradiated samples with UV light at 310 nm wavelength for 2 h. After the UV irradiation, we washed the DNA origami with Amicon 100K filter tubes, using the buffer containing 5 mM MgCl_2_, 5 mM NaCl, 5 mM Tris and 1 mM EDTA, four rounds (7,000*g* for 1.5 min per round).

#### Peptide–ssDNA conjugate

We purchased ssDNA with a dibenzocyclooctyne modification at its 5′ end from Biomers (Supplementary Data). The conjugation of the peptide to the DNA was performed via copper-free click chemistry in 1× PBS. In the reaction, the molar ratio of peptide to ssDNA was 50 to 1. We purified the conjugate by using a proFIRE system (Dynamic Biosensors).

#### The affinity of the peptide–ssDNA conjugate to DR5

The binding kinetics of the peptide–ssDNA conjugates to DR5 were studied by SPR using a Biacore T200 instrument (GE Healthcare). First, streptavidin (Sigma-Aldrich, S4762) was dissolved in 10 mM sodium acetate pH 4.5 and immobilized on a CM3 chip (GE Healthcare, BR100536) via amine coupling following the manufacturer’s instructions. The biotinylated anchor oligonucleotides, complementary to the peptide–ssDNA conjugates, were captured at 150 RU by injecting them over the streptavidin surface for 660 s at a flow rate of 10 µl min^−1^, followed by a 60 s wash with running buffer 1× HBS-EP+ (GE Healthcare, BR100669). The peptide–ssDNA conjugates were immobilized by DNA complementarity at 300 RU by injecting them at a flow rate of 5 µl min^−1^ and washing the excess of conjugates for 60 s at 30 µl min^−1^ with the same running buffer. As a negative control, a parallel flow cell was modified with biotinylated anchors but without peptide–ssDNA. The DR5 analyte (R&D Systems, 10140-T2) was diluted from 516 nM to 32.25 nM and injected as a single-cycle kinetics experiment, with a flow rate of 30 µl min^−1^, and 500 s contact time for each concentration. After the last concentration, the dissociation phase lasted 1,000 s at the same flow rate, and the surface was regenerated with two 30 s injections of 50 mM NaOH. The single-cycle kinetics runs were carried out in HBS-EP+ buffer at pH 7.4 or 6.5, for both the empty negative flow cell and the peptide–ssDNA modified flow cell. The sensogram data were analysed with the BIA-evaluation 3.0 software assuming a 1:1 Langmuir binding model.

#### The affinity of the peptide origami to DR5

This interaction was also analysed by SPR. As described for the peptide–ssDNA conjugates, streptavidin and biotinylated oligonucleotides, complementary to the anchoring oligonucleotides in the origami, were immobilized on a CM3 chip. By DNA complementarity, the surface was saturated with the peptide origami at around 600 RU by injecting it at flow rate of 10 µl min^−1^, and washing for 60 s at 30 µl min^−1^ with the same running buffer. As a reference sample, a parallel reference flow cell was saturated with empty origami. The DR5 analyte (R&D Systems, 10140-T2) was diluted from 1,032 nM to 64.5 nM and injected as a single-cycle kinetics experiment, with a flow rate of 30 µl min^−1^, and 500 s contact time for each concentration. After the last concentration, the dissociation phase lasted 1,000 s at the same flow rate, and the surface was regenerated with two 30 s injections of 50 mM NaOH. Three replicates of the single-cycle kinetics were run with HBS-EP+ buffer supplemented with 5 mM MgCl_2_ at pH 7.4, for both the peptide origami and the empty origami flow cells. The sensogram data were analysed with the BIA-evaluation 3.0 software assuming a 1:1 Langmuir binding model.

#### Peptide–ssDNA conjugate and PEG_5K_–ssDNA hybridization to DNA origami

A 20-fold excess of conjugate to each mini-scaffold on the DNA origami was mixed with UV-irradiated DNA origami. We kept the mixture under 37 °C for 1 h followed by room temperature overnight. To wash away the free peptide–ssDNA conjugate from DNA origami, we used Amicon 100K filter tubes, with six rounds of dilution and concentration using 1× PBS (7,000 rcf for 1.5 min per round). To modify the surface of the origami with a certain number of PEG_5K_, we added an excess of PEG_5K_–ssDNA polymers to the origami with ssDNA protrusions on its surface (the molar ratio of ssDNA–PEG_5K_ to each protrusion on the origami was 5 to 1). The structures were finally purified with 1× PBS using Amicon 100K filter tubes.

### DNA origami characterization

#### Gel electrophoresis

We prepared 2% agarose gels in 0.5× Tris-borate-EDTA (TBE) buffer supplemented with 10 mM MgCl_2_ and 0.5 mg ml^−1^ ethidium bromide. On an ice bath, we ran electrophoresis in 0.5× TBE buffer supplemented with 10 mM MgCl_2_ at 90 V for 3 h. Gels were imaged under a GE LAS 4000 imager.

#### Negative-stain TEM

We mixed 8 µl of 1 M NaOH with 400 µl of 2% (w/v) uranyl formate. The mixture was then immediately centrifuged at 16,500*g* for 5 min. We added 5 µl of 5 nM DNA origami to a glow-discharged, carbon-coated, formvar resin grid for 1 min. We then blotted the sample drop away with filter paper before we applied 5 µl of uranyl formate solution to the grid for 40 s. The sample was air-dried and imaged in a 120 kV LaB6 microscope (Talos 120C G2 with Ceta-D detector) at different magnifications. The particle class-averaging processing was carried out in Scipion.

#### Cryo-EM

Vitrobot Mk4 (FEI) was used to prepare cryo specimens. We used the Quantifoil R 1.2/1.3 (LF, 200 Mesh, Gold) grid. The grid was glow-discharged under 25 mA for 30 s. We applied 3 µl of the highly concentrated (>500 nM) DNA origami solution to the grid. The grids were incubated at a relative humidity of 100% for 1–5 min and frozen in liquid ethane after blotting (3 s, drain time 1 s). The grids were kept in liquid nitrogen until imaging. Cryo-EM data were collected with EPU software using a Krios G3i TEM operated at 300 kV. Images were acquired in 81 kx nanoprobe EFTEM SA mode with a slit width of 10 eV using a K3 Bioquantum. Exposure time was 4.4 s, during which 45 movie frames were collected with a fluency of 0.90 e Å^−2^ per frame. Image pre-processing until particle picking was carried out in Warp^45^, with subsequent processing using CryoSPARC^34^. An outline of the data processing pipeline can be found in Supplementary Fig. 17. For density visualization only, the reconstructed map was post-processed using DeepEMhancer^46^ using tight mask target settings and dust hiding was performed using UCSF ChimeraX^47^. A comparison between the original and post-processed maps can be found in Supplementary Fig. 11.

#### Stability of the origami in cell culture medium

Five microlitres of 100 nM origami was mixed with 95 μl of Dulbecco’s modified Eagle medium (DMEM) containing 10% foetal bovine serum (FBS). The samples were kept in a humidified incubator containing 5% CO_2_ at 37 °C. At different timepoints, the samples were checked via 2% agarose gel electrophoresis in 0.5× TBE buffer supplemented with 10 mM MgCl_2_ at 90 V for 3 h. Gels were imaged under a GE LAS 4000 imager.

#### Quantification of mini-scaffold and peptide on origami

Origami structures containing different numbers of peptides were incubated with the Cy5-labelled oligonucleotides complementary to the mini-scaffold. The molar ratio of the Cy5-labelled oligo to each mini-scaffold of origami was 20 to 1. After purification, concentrations of origami samples were set to 10 nM. Each sample’s Cy5 signal of 100 µl was measured via a multimode microplate reader (Varioskan LUX).

### Coarse-grained molecular dynamics simulation of the origami

The origami design file from Cadnano was converted to oxDNA format using tacoxDNA^48^. The oxDNA simulation started with a relaxation step for removing possible overlapping nucleotides, followed by a molecular dynamics simulation run to reduce overstretched bonds. Afterward, the structure was simulated for 1 × 10^8^ steps, with a time step of 0.005 oxDNA time units. The simulations were run with the oxDNA2 model at 30 °C with a salt concentration of 0.15 M and an Anderson-like thermostat. Every 2 × 10^4^ steps were saved as one simulation state. The mean structure was calculated using the oxDNA analysis tool^29,30^.

### Cell experiments

#### Cell culture

Immortalized human cell lines, including SK-BR-3 (ATCC, HTB-30), HBEC-5i (ATCC, CRL-3245) and HEK293 (ATCC, CRL-1573), were purchased. SK-BR-3 cells were cultured in DMEM high glucose (Sigma-Aldrich, D5796) containing 10% FBS (Sigma-Aldrich, F2442) and 100 units ml^−1^ penicillin–streptomycin (Sigma-Aldrich, P4333) in a humidified incubator containing 5% CO_2_ at 37 °C. For the culture of HEK293, Eagle’s Minimum Essential Medium (ATCC, 30-2003) supplemented with 10% FBS and 100 units ml^−1^ penicillin–streptomycin was used; for the culture of HEEC-5i, the flasks or plates were pre-coated with 0.1% gelatin (ATCC, PCS-999-027) for 1 h. Cells were then seeded on the flasks and cultured in DMEM: F12 (ATCC, 30-2006) supplemented with 40 µg ml^−1^ endothelial growth supplement (Sigma, E2759) and 10% FBS.

#### Confocal assay

A total of 2.5 × 10^4^ cells per well were seeded in an eight-well Millicell EZ SLIDE for 24 h. Before adding origami, the medium was replaced by fresh medium with a pH of 7.4 or 6.5. The final concentration of Cy5-labelled origami in 0.5 ml of medium was 5 nM. After a 4 h incubation in a humidified incubator containing 5% CO_2_ at 37 °C, cells were fixed with 4% formaldehyde. To detect the DR5, the cells were incubated with 66 nM human TRAIL R2/TNFRSF10B antibody (R&D Systems, MBA6311) overnight at 4 °C. The next day, goat anti-mouse IgG H&L (Alexa Fluor 488; Abcam, ab150113), at a concentration of 2 µg ml^−1^, was used as the secondary antibody to visualize the bound primary antibody against DR5. After staining with Fluoroshield Mounting Medium with 4′,6-diamidino-2-phenylindole (DAPI; Abcam, ab104139), we imaged the cells under LSM700 (Zeiss).

#### Flow cytometry

For the quantitative assay of origami binding to cells, 1 × 10^7^ SK-BR-3 cells per well were cultured in six-well plates overnight. The medium of each well was replaced by 2 ml of fresh medium with pH 7.4 or 6.5 containing 5 nM of Cy5-labelled origami structures for 4 h treatments in a humidified incubator containing 5% CO_2_ at 37 °C. Cells were collected and washed twice with cold 1× PBS, followed by a flow cytometry measurement. For the cell apoptosis assay, with six-well plates, 1 × 10^7^ SK-BR-3 cells were seeded in each well for culture overnight. The medium was removed, and 2 ml of fresh medium with pH 7.4 or 6.5 containing 5 nM origami was added to the cells for 24 h treatments. All cells (including detached cells in the medium) were collected and resuspended in cold 1× PBS. Cells were then stained with annexin V-fluorescein isothiocyanate (FITC) and propidium iodide (PI), sequentially, according to the protocol of a commercial apoptosis assay kit (Thermo Fisher Scientific, V13242). Cells were analysed using the Cytek Aurora flow cytometer.

#### Origami quantification with repair qPCR

A total of 1 × 10^7^ SK-BR-3 cells per well were cultured in four-well plates overnight. The medium was replaced by 2 ml of fresh medium with pH 7.4 or 6.5 containing 5 nM origami structures for 4 h treatments. The cell layer in each well was gently washed with cold 1× PBS twice, which was followed with or without 5 U μl^−1^ Benzonase (Invitrogen) treatment. After cell washing and lysing using 100 μl DNAzol Reagent (Life Technologies), DNA pellets were collected according to a previously established protocol^38^. In 1× PBS, the DNA samples were denatured under 95 °C for 30 min. Then, the PreCR Repair Mix (New England Biolabs) was used according to the kit protocol to repair the DNA samples. The rationale for this step is that the structures were both UV-cross-linked and subjected to very high temperatures. The Luna Universal qPCR Master Mix (New England Biolabs) and the primer pair (forward: CTGGCTCGAAAATGCCTCT, reverse: ACCAGTATAAAGCCAACGCT) were used together to quantify the scaffold of origami in the samples.

#### Cell viability assay

A total of 5 × 10^4^ cells per well were seeded on 96-well opaque white polystyrene microplates (Fisher Scientific, 10583534) and cultured for 24 h. The cell culture medium was replaced with 95 μl of fresh medium with pH 7.4 or 6.5, followed by the addition of 5 μl of 100 nM DNA origami. At different timepoints, the plate was transferred out of the culture incubator and incubated at room temperature for 20 min. To measure the cell viability, the CellTiter-Glo Luminescent Cell Viability Assays (Promega, G7571) were used. The luminescence was recorded on a multimode microplate reader (Varioskan LUX). The luminescence of the wells containing medium without cells was used as the background control. The following equation was used to calculate the relative cell viability: % viable cells = (luminescence_sample_ − luminescence_background_)/(luminescence_PBS_ − luminescence_background_) × 100.

### Animal experiments

#### Ethics permit

All animal handling and experimental procedures were carried out according to local ethics guidelines and approved by Stockholm’s Animal Experimentation Ethics Committee (Stockholms djurförsöksetiska nämnd, Dnr 16041-2019).

#### Mouse model

Mice were ordered from Charles River and bred at the animal facility Comparative Medicine Biomedicum (KMB), Solna Campus, Karolinska Institutet. Female BALB/c Nude CByJ.Cg-Foxn1nu/J mice at the age of 29 days were used to establish the SK-BR-3 xenograft model. A total of 5 × 10^6^ SK-BR-3 cells were subcutaneously inoculated into the upper left flank of mice in 0.1 ml of Matrigel (Corning, 354234) diluted in cold PBS (Matrigel:PBS, 1:1). After 1 week, mice were randomized into treatment groups for experiments.

#### Biodistribution

The Alexa750-labelled origami was administered to the mice intravenously into the tail (100 μl, 100 nM) or intratumourally (20 μl, 200 nM). To track the biodistribution of the origami, the live mice were imaged at different timepoints using a PerkinElmer in vivo imaging system.

#### Anti-tumour efficacy assay

PBS or origami (100 μl of 100 nM origami for i.v. injection; 20 μl of 200 nM origami for i.t. injection) was administrated according to the therapy regimes. We measured the sizes of tumours after each injection with a caliper and calculated the tumour volume using the following equation: tumour volume (mm^3^) = (length × width^2^) × ½.

### Statistics and reproducibility

For quantitative analysis, a minimum of three biological replicates were analysed excluding the in vivo data of PBS (*n* = 2). Data were analysed by ordinary one-way analysis of variance (ANOVA) with Tukey post-tests and Student’s *t*-test. A *P* value <0.05 was considered statistically significant. No statistical method was used to pre-determine sample size. No data were excluded from the analyses. The investigators were not blinded to allocation during experiments and outcome assessment.

### Reporting summary

Further information on research design is available in the Nature Portfolio Reporting Summary linked to this article.

## Online content

Any methods, additional references, Nature Portfolio reporting summaries, source data, extended data, supplementary information, acknowledgements, peer review information; details of author contributions and competing interests; and statements of data and code availability are available at 10.1038/s41565-024-01676-4.

## Supplementary information

Supplementary Information Supplementary Figs. 1–22 and uncropped gel images.

Reporting Summary

Supplementary Data DNA oligo sequences of the origami.

## Source data

Source Data Fig. 1 Statistical source data.

Source Data Fig. 2 Statistical source data and uncropped gel images.

Source Data Fig. 3 Statistical source data.

Source Data Fig. 5 Statistical source data.

Source Data Fig. 6 Statistical source data.

Source Data Supplementary Fig. 4 Statistical source data.

Source Data Supplementary Fig. 12 Statistical source data.

Source Data Supplementary Fig. 13 Statistical source data.

Source Data Supplementary Fig. 18 Statistical source data.

Source Data Supplementary Fig. 19 Statistical source data.

Source Data Supplementary Fig. 20 Statistical source data.

Source Data Supplementary Fig. 21 Statistical source data.

Source Data Supplementary Fig. 22 Statistical source data.

## Data Availability

The DNA origami design is available in the Supplementary Information as well as deposited to nanobase.org with accession number 233 at https://nanobase.org/structure/233. The electron density maps of the UV-cross-linked robotic switch are available in the Electron Microscopy Data Bank (EMDB) as entry EMD-19129. Source data are provided with this paper.

## References

[1] Foy, S. P. et al. Non-viral precision T cell receptor replacement for personalized cell therapy. *Nature* **615**, 687–696 (2023). 36356599 10.1038/s41586-022-05531-1 PMC9768791

[2] Choe, J. H. et al. SynNotch-CAR T cells overcome challenges of specificity, heterogeneity, and persistence in treating glioblastoma. *Sci. Transl. Med.* **13**, 7378 (2021). 10.1126/scitranslmed.abe7378 PMC8362330 33910979

[3] Twohig, J. P., Cuff, S. M., Yong, A. A. & Wang, E. C. Y. The role of tumor necrosis factor receptor superfamily members in mammalian brain development, function and homeostasis. *Rev. Neurosci.* **22**, 509–533 (2011). 21861782 10.1515/RNS.2011.041 PMC3460212

[4] von Karstedt, S., Montinaro, A. & Walczak, H. Exploring the TRAILs less travelled: TRAIL in cancer biology and therapy. *Nat. Rev. Cancer* **17**, 352–366 (2017). 28536452 10.1038/nrc.2017.28

[5] Ward-Kavanagh, L. K., Lin, W. W., Šedý, J. R. & Ware, C. F. The TNF receptor superfamily in co-stimulating and co-inhibitory responses. *Immunity* **44**, 1005–1019 (2016). 27192566 10.1016/j.immuni.2016.04.019 PMC4882112

[6] Ashkenazi, A. Targeting death and decoy receptors of the tumour-necrosis factor superfamily. *Nat. Rev. Cancer* **2**, 420–430 (2002). 12189384 10.1038/nrc821

[7] Aggarwal, B. B. Signalling pathways of the TNF superfamily: a double-edged sword. *Nat. Rev. Immunol.* **3**, 745–756 (2003). 12949498 10.1038/nri1184

[8] Pan, L. et al. Higher-order clustering of the transmembrane anchor of DR5 drives signaling. *Cell* **176**, 1477–1489.e14 (2019). 30827683 10.1016/j.cell.2019.02.001 PMC6529188

[9] Vanamee, É. S. & Faustman, D. L. Structural principles of tumor necrosis factor superfamily signaling. *Sci. Signal* **11**, eaao4910 (2018). 29295955 10.1126/scisignal.aao4910

[10] Graves, J. D. et al. Apo2L/TRAIL and the death receptor 5 agonist antibody AMG 655 cooperate to promote receptor clustering and antitumor activity. *Cancer Cell* **26**, 177–189 (2014). 25043603 10.1016/j.ccr.2014.04.028

[11] Gieffers, C. et al. APG350 induces superior clustering of TRAIL receptors and shows therapeutic antitumor efficacy independent of cross-linking via Fcγ receptors. *Mol. Cancer Ther.* **12**, 2735–2747 (2013). 24101228 10.1158/1535-7163.MCT-13-0323

[12] Schneider, H. et al. TRAIL-inspired multivalent dextran conjugates efficiently induce apoptosis upon DR5 receptor clustering. *ChemBioChem* **20**, 3006–3012 (2019). 31206933 10.1002/cbic.201900251

[13] Lamanna, G. et al. Multimerization of an apoptogenic TRAIL-mimicking peptide by using adamantane-based dendrons. *Chemistry* **19**, 1762–1768 (2013). 23239456 10.1002/chem.201202415

[14] Moyer, T. J. et al. Self-assembled peptide nanostructures targeting death receptor 5 and encapsulating paclitaxel as a multifunctional cancer therapy. *ACS Biomater. Sci. Eng.* **5**, 6046–6053 (2019). 33304996 10.1021/acsbiomaterials.9b01259 PMC7725269

[15] Swers, J. S. et al. Multivalent scaffold proteins as superagonists of TRAIL receptor 2-induced apoptosis. *Mol. Cancer Ther.* **12**, 1235–1244 (2013). 23645592 10.1158/1535-7163.MCT-12-1107

[16] Ma, N. et al. Nanoscale organization of TRAIL trimers using DNA origami to promote clustering of death receptor and cancer cell apoptosis. *Small* **19**, e2206160 (2023). 36890776 10.1002/smll.202206160

[17] Berger, R. M. L. et al. Nanoscale FasL organization on DNA origami to decipher apoptosis signal activation in cells. *Small* **17**, e2101678 (2021). 34057291 10.1002/smll.202101678

[18] Wang, Y., Baars, I., Fördös, F. & Högberg, B. Clustering of death receptor for apoptosis using nanoscale patterns of peptides. *ACS Nano* **15**, 9614–9626 (2021). 34019379 10.1021/acsnano.0c10104 PMC8223489

[19] Uberti, D. et al. Blockade of the tumor necrosis factor-related apoptosis inducing ligand death receptor DR5 prevents beta-amyloid neurotoxicity. *Neuropsychopharmacology* **32**, 872–880 (2007). 16936710 10.1038/sj.npp.1301185

[20] Solà-Riera, C. et al. Hantavirus inhibits TRAIL-mediated killing of infected cells by downregulating death receptor 5. *Cell Rep.* **28**, 2124–2139.e6 (2019). 31433987 10.1016/j.celrep.2019.07.066

[21] Mak, T. W. & Yeh, W.-C. Signaling for survival and apoptosis in the immune system. *Arthritis Res.* **4**, S243–S252 (2002). 12110144 10.1186/ar569 PMC3240145

[22] Lu, H. et al. On-demand targeting nanotheranostics with stimuli-responsive releasing property to improve delivery efficiency to cancer. *Biomaterials* **290**, 121852 (2022). 36270058 10.1016/j.biomaterials.2022.121852

[23] Kuzyk, A., Urban, M. J., Idili, A., Ricci, F. & Liu, N. Selective control of reconfigurable chiral plasmonic metamolecules. *Sci. Adv.* **3**, e1602803 (2017). 28439556 10.1126/sciadv.1602803 PMC5400443

[24] Ng, C. et al. Folding double-stranded DNA into designed shapes with triplex-forming oligonucleotides. *Adv. Mater.* **35**, e2302497 (2023). 37311656 10.1002/adma.202302497

[25] Sachenbacher, K. et al. Triple-stranded DNA as a structural element in DNA origami. *ACS Nano* **17**, 9014–9024 (2023). 37159224 10.1021/acsnano.2c11402 PMC10210531

[26] Ijäs, H., Hakaste, I., Shen, B., Kostiainen, M. A. & Linko, V. Reconfigurable DNA origami nanocapsule for pH-controlled encapsulation and display of cargo. *ACS Nano* **13**, 5959–5967 (2019). 30990664 10.1021/acsnano.9b01857 PMC7076726

[27] Williamson, P., Ijäs, H., Shen, B., Corrigan, D. K. & Linko, V. Probing the conformational states of a pH-sensitive DNA origami zipper via label-free electrochemical methods. *Langmuir* **37**, 7801–7809 (2021). 34128683 10.1021/acs.langmuir.1c01110 PMC8280702

[28] Julin, S., Linko, V. & Kostiainen, M. A. Reconfigurable pH-responsive DNA origami lattices. *ACS Nano* **17**, 11014–11022 (2023). 37257137 10.1021/acsnano.3c03438 PMC10278177

[29] Poppleton, E. et al. Design, optimization and analysis of large DNA and RNA nanostructures through interactive visualization, editing and molecular simulation. *Nucleic Acids Res.* **48**, e72 (2020). 32449920 10.1093/nar/gkaa417 PMC7337935

[30] Bohlin, J. et al. Design and simulation of DNA, RNA and hybrid protein–nucleic acid nanostructures with oxView. *Nat. Protoc.* **17**, 1762–1788 (2022). 35668321 10.1038/s41596-022-00688-5

[31] Gür, F. N. et al. Double- to single-strand transition induces forces and motion in DNA origami nanostructures. *Adv. Mater.* **33**, e2101986 (2021). 34337805 10.1002/adma.202101986 PMC7611957

[32] Rajendran, A., Endo, M., Katsuda, Y., Hidaka, K. & Sugiyama, H. Photo-cross-linking-assisted thermal stability of DNA origami structures and its application for higher-temperature self-assembly. *J. Am. Chem. Soc.* **133**, 14488–14491 (2011). 21859143 10.1021/ja204546h

[33] Gerling, T., Kube, M., Kick, B. & Dietz, H. Sequence-programmable covalent bonding of designed DNA assemblies. *Sci. Adv.* **4**, eaau1157 (2018). 30128357 10.1126/sciadv.aau1157 PMC6097813

[34] Punjani, A., Rubinstein, J. L., Fleet, D. J. & Brubaker, M. A. cryoSPARC: algorithms for rapid unsupervised cryo-EM structure determination. *Nat. Methods* **14**, 290–296 (2017). 28165473 10.1038/nmeth.4169

[35] Punjani, A. & Fleet, D. J. 3DFlex: determining structure and motion of flexible proteins from cryo-EM. *Nat. Methods* **20**, 860–870 (2023). 37169929 10.1038/s41592-023-01853-8 PMC10250194

[36] Suk, J. S., Xu, Q., Kim, N., Hanes, J. & Ensign, L. M. PEGylation as a strategy for improving nanoparticle-based drug and gene delivery. *Adv. Drug Deliv. Rev.* **99**, 28–51 (2016). 26456916 10.1016/j.addr.2015.09.012 PMC4798869

[37] Ponnuswamy, N. et al. Oligolysine-based coating protects DNA nanostructures from low-salt denaturation and nuclease degradation. *Nat. Commun.* **8**, 15654 (2017). 28561045 10.1038/ncomms15654 PMC5460023

[38] Okholm, A. H. et al. Quantification of cellular uptake of DNA nanostructures by qPCR. *Methods* **67**, 193–197 (2014). 24472874 10.1016/j.ymeth.2014.01.013

[39] Chen, J. H. & Seeman, N. C. Synthesis from DNA of a molecule with the connectivity of a cube. *Nature* **350**, 631–633 (1991). 2017259 10.1038/350631a0

[40] Rothemund, P. W. K. Folding DNA to create nanoscale shapes and patterns. *Nature* **440**, 297–302 (2006). 16541064 10.1038/nature04586

[41] Knappe, G. A., Wamhoff, E.-C. & Bathe, M. Functionalizing DNA origami to investigate and interact with biological systems. *Nat. Rev. Mater.* **8**, 123–138 (2023). 37206669 10.1038/s41578-022-00517-x PMC10191391

[42] Ducani, C., Kaul, C., Moche, M., Shih, W. M. & Högberg, B. Enzymatic production of ‘monoclonal stoichiometric’ single-stranded DNA oligonucleotides. *Nat. Methods* **10**, 647–652 (2013). 23727986 10.1038/nmeth.2503 PMC3843646

[43] Praetorius, F. et al. Biotechnological mass production of DNA origami. *Nature* **552**, 84–87 (2017). 29219963 10.1038/nature24650

[44] Wagenbauer, K. F. et al. How we make DNA origami. *ChemBioChem* **18**, 1873–1885 (2017). 28714559 10.1002/cbic.201700377

[45] Tegunov, D. & Cramer, P. Real-time cryo-electron microscopy data preprocessing with Warp. *Nat. Methods* **16**, 1146–1152 (2019). 31591575 10.1038/s41592-019-0580-y PMC6858868

[46] Sanchez-Garcia, R. et al. DeepEMhancer: a deep learning solution for cryo-EM volume post-processing. *Commun. Biol.* **4**, 874 (2021). 34267316 10.1038/s42003-021-02399-1 PMC8282847

[47] Pettersen, E. F. et al. UCSF ChimeraX: structure visualization for researchers, educators, and developers. *Protein Sci.* **30**, 70–82 (2021). 32881101 10.1002/pro.3943 PMC7737788

[48] Suma, A. et al. TacoxDNA: a user-friendly web server for simulations of complex DNA structures, from single strands to origami. *J. Comput. Chem.* **40**, 2586–2595 (2019). 31301183 10.1002/jcc.26029

