# Supplementary material for: A DNA robotic switch with regulated autonomous display of cytotoxic ligand nanopatterns
Source: Nat Nanotechnol. 2024 Jul 1;19(9):1366–74. doi: 10.1038/s41565-024-01676-4 (PMC11405282; doi:10.1038/s41565-024-01676-4)

nature nanotechnology

Supplementary information https://doi.org/10.1038/s41565-024-01676-4

A DNA robotic switch with regulated autonomous display of cytotoxic ligand nanopatterns

In the format provided by the authors and unedited

Table of Contents

1. Origami design 2

2. Mean structure from oxDNA simulation 3

3. Calculation consideration on distance from peptide to origami surface 4

4. Distances from the peptide to the origami surface 5

5. Folding screening 6

6. Gel electrophoresis of origami before and after UV irradiation 7

7. Negative-stain TEM image of the origami before UV irradiation in 5mM $MgCl_2$.......... 8

8. Negative-stain TEM image of the origami before UV irradiation (buffer exchanged from 5 mM $MgCl_2$ into 1X PBS) 9

9. Negative-stain TEM image of UV-irradiated origami in 5 mM $MgCl_2$.......... 10

10. Negative-stain TEM image of UV-irradiated origami in 1X PBS 11

11. Cryo-EM dataset pre-processing and processing workflow 12

12. Mini-Scaffold quantification of the UV-crosslinked origami 13

13. FRET effects of the origami containing only one DNA triplex. 14

14. Conjugation of the peptide with DNA 15

15. The affinity of the peptide-DNA conjugate to DR5 under different pH 16

16. Peptide abundance assay on gel 17

17. The affinity of the origami displaying a peptide pattern to DR5 18

18. Cell interaction of the origami 19

19. Origami quantification per cell using repair qPCR 20

20. Tumor growth of the mice treated via intravenous injection 21

21. Tumor growth of the mice treated via intratumoral injection 21

22. Cleaved caspase-3 and 8 levels in the tumors receiving intratumoral injection 22

1. Origami design

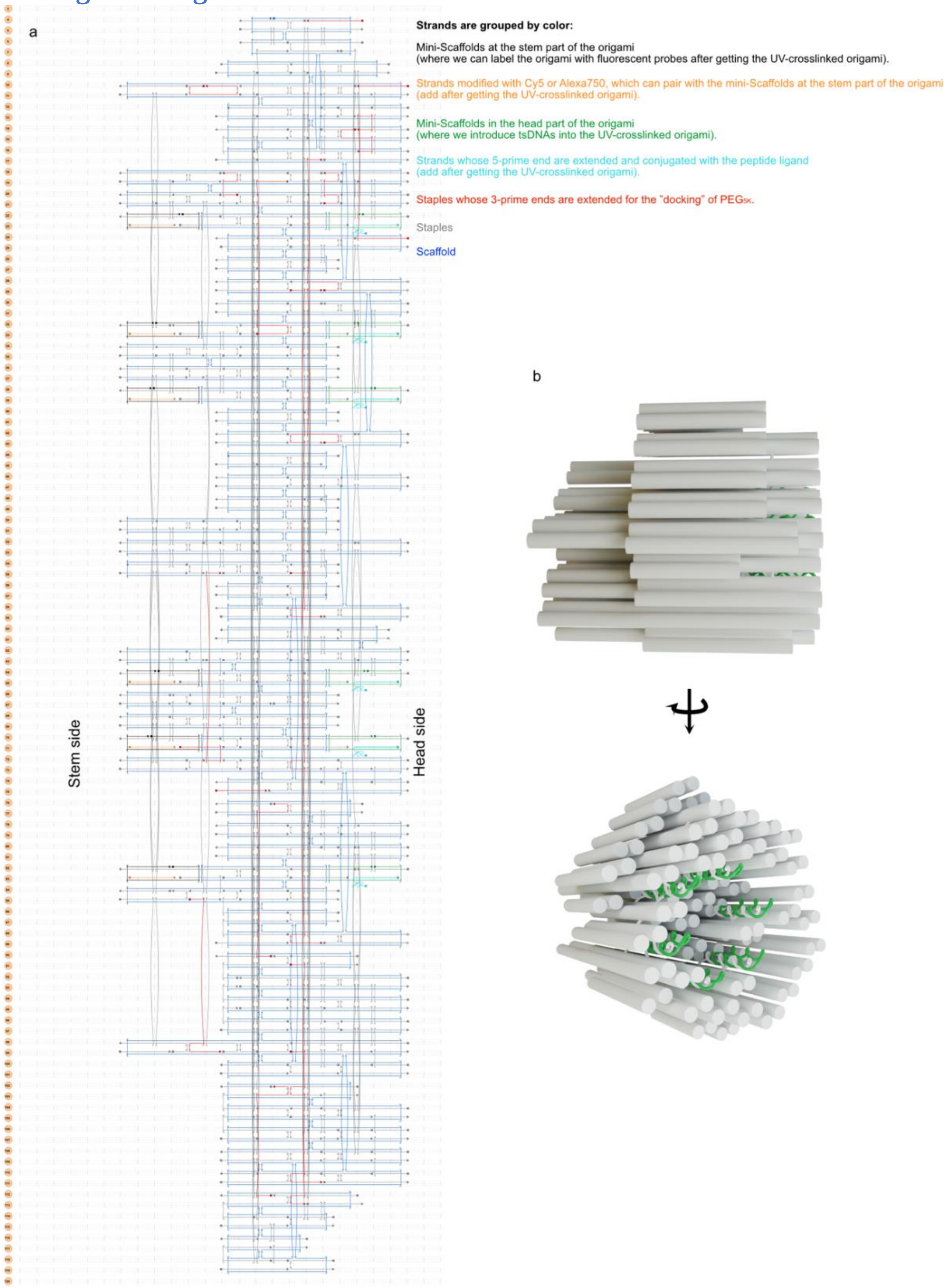

Supplementary Fig.1: The origami design blueprint in Cadnano (a) and the 3D rendering of the designed origami (b).

2. Mean structure from oxDNA simulation

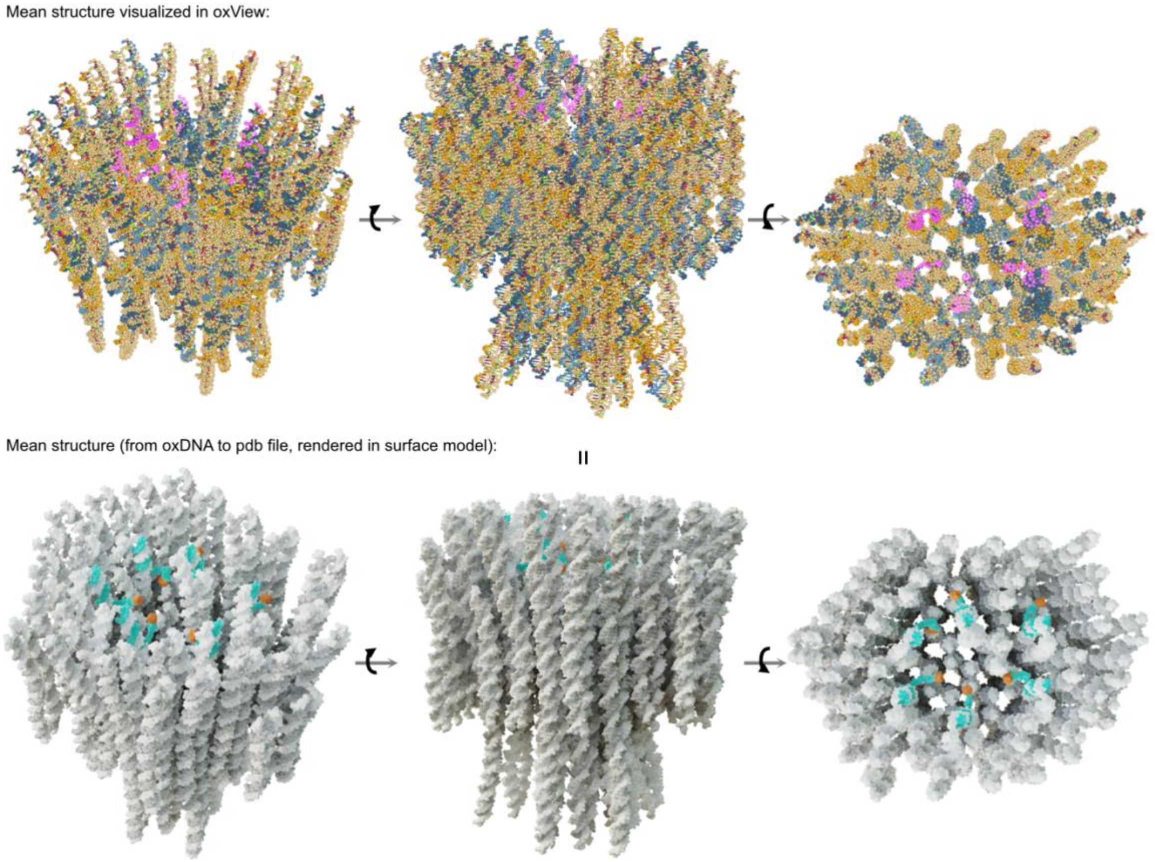

Supplementary Fig.2: The mean origami structure computed from oxDNA simulation. Structures at the up panel are the screenshots of different views in oxView. Structures at the bottom panel are the re-rendered mean structure for better visualization, which was achieved by converting the mean structure into its pdb format first, and then the pdb file of the structure was rendered as its surface model.

3. Calculation consideration on distance from peptide to origami surface

To determine whether the terminus of each TFO strand is inside or outside the cavity of DNA origami, their distances to the boundary plane of the cavity are calculated. We defined the boundary plane by three nucleotides at the entrance of the cavity (The IDs of the nucleotides in oxDNA topology are: **P1**: 10231, **P2**: 14721, **P3**:15693) as shown:

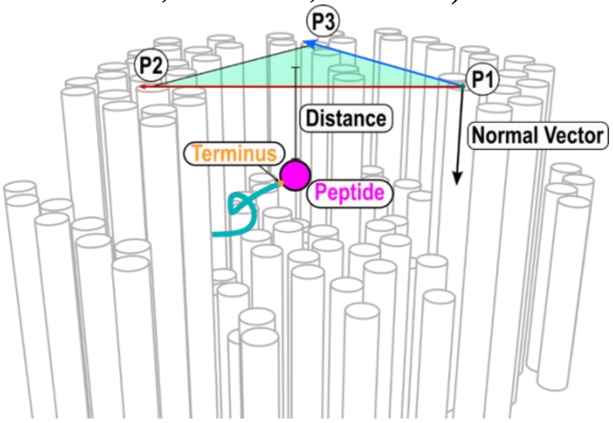

The coordinates of the three plane-defining nucleotides and the termini of the six TFOs were extracted from the oxDNA simulation trajectory file using the script subset_trajectory from the oxDNA Analysis Tools package [Nucleic Acids Research, Volume 48, Issue 12, Page e72 (2020)]. For each frame in the simulation, first, the two vectors defined by the three points were obtained:

$\mathbf{V_1}$ = P2 - P1 = $(x_2 - x_1, y_2 - y_1, z_2 - z_1)$

$\mathbf{V_2}$ = P3 - P1 = $(x_3 - x_1, y_3 - y_1, z_3 - z_1)$

Then calculate the cross product (normal vector) of V1 and V2 as the normal vector of the plane:

Normal Vector $\mathbf{N} = \mathbf{V_1} \times \mathbf{V_2} = (A, B, C)$

$A = (y_2 - y_1)(z_3 - z_1) - (z_2 - z_1)(y_3 - y_1)$

$B = (z_2 - z_1)(x_3 - x_1) - (x_2 - x_1)(z_3 - z_1)$

$C = (x_2 - x_1)(y_3 - y_1) - (y_2 - y_1)(x_3 - x_1)$

Find the constant D using the plane equation and one of the points on the plane (for example, P1):

$D = -(Ax_1 + By_1 + Cz_1)$

So, the equation of the plane is:

$Ax + By + Cz + D = 0.$

Calculate the distance from the each point $Q_i(x_i, y_i, z_i)$ to the plane using the distance formula:

$d_i = |Ax_i + By_i + Cz_i + D| / \text{sqrt}(A^2 + B^2 + C^2)$

Plug in the coordinates of terminus point Q_i and the coefficients A, B, C, and constant D into the formula to get the distance from point Q_i to the plane. To determine whether the terminus is on the same side of the plane as the normal vector, plug in the points to the plane equation:

On the same side: if $Ax_i + By_i + Cz_i + D > 0$

On the same side: if $Ax_i + By_i + Cz_i + D > 0$

On the plane: if $Ax_i + By_i + Cz_i + D = 0$

Finally, we defined the signed distance $\mathbf{d}_i$ as the product of the distance and the sign of $(Ax_i + By_i + Cz_i + D)$.

Taking into consideration of the peptide size

Furthermore, to make the estimation closer to the reality, we took the von Der Waals diameter of the peptide (2.8 nm) into consideration. To calculate the distance correction due to the size of the peptide, we first found out the backbone direction of the terminus nucleotide which directly connected to the peptide. It happens to be the $\mathbf{a_3}$ vector of the nucleotide in oxDNA configuration file. Then we obtained the angle θ between the $\mathbf{a_3}$ vector and the normal vector

of the boundary plane **N**. Finally, based on the geometry as shown in Fig. SY, the corresponding corrected distance can be obtained with the formula:

$$d' = d - R\,(1 - \cos\theta)$$

where d' is the corrected distance between the peptide to the boundary plane, d is the distance between the terminus of the TFO to the boundary plane, R is the radius of the peptide, which is 1.4 nm, θ is the angle between **N** and $\mathbf{a_3}$.

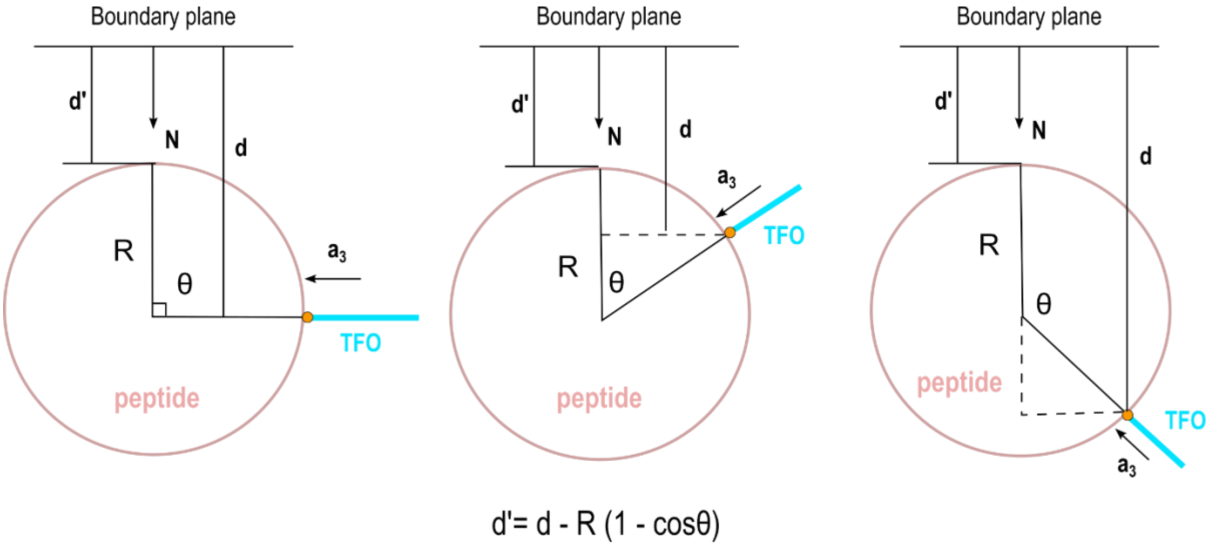

Supporting Fig.3: The peptide size and orientation information of the base that connects the peptide are included for the distance calculation.

4. Distances from the peptide to the origami surface

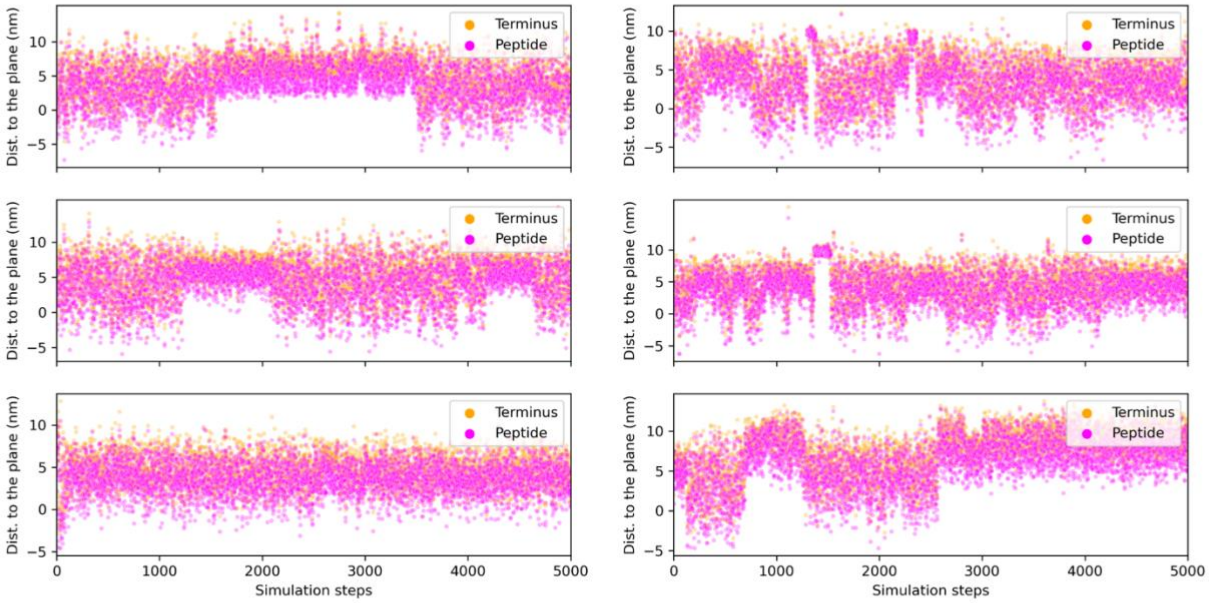

Supplementary Fig.4: Real-time distances, from the TFO terminus (dots in orange) or from the peptide surface to the origami surface (dots in magenta, recalculated from the dataset of orange points via including the orientation information of the TFO terminus and the physical diameter of the peptide), along the simulation.

5. Folding screening

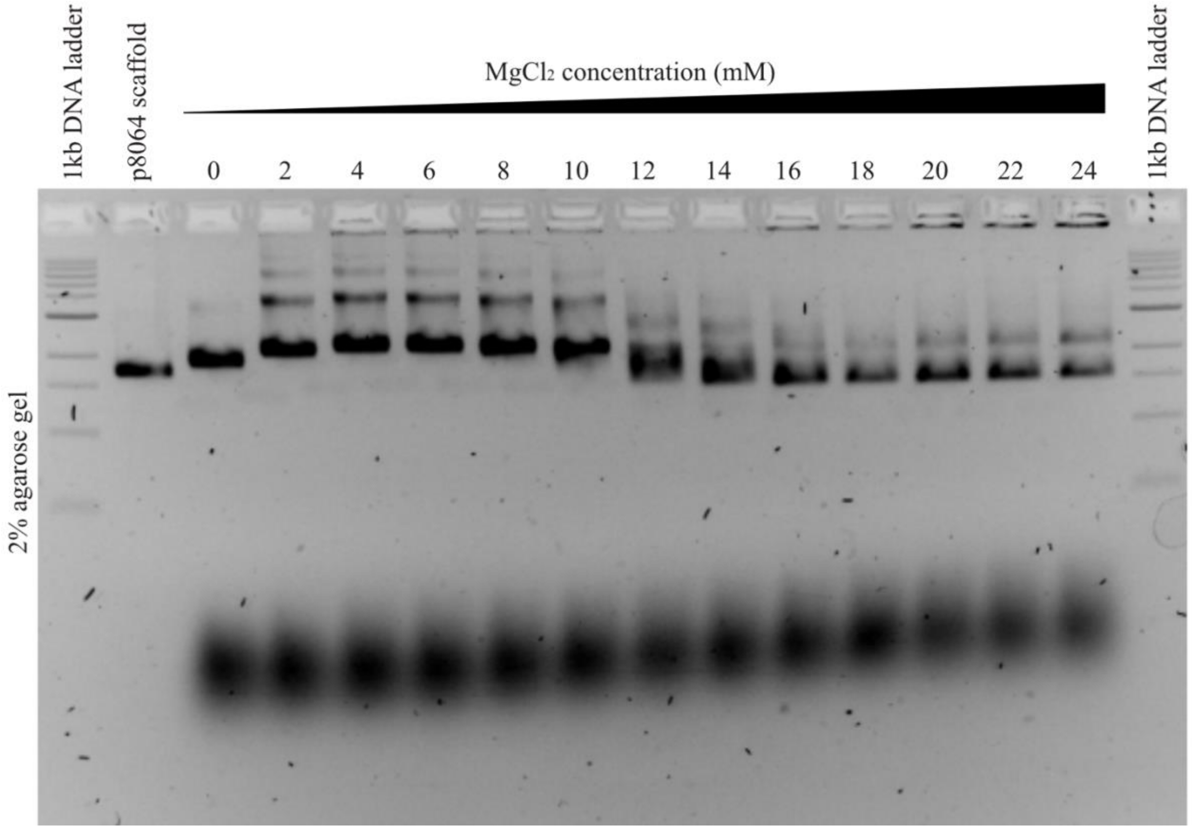

Supplementary Fig.5: $MgCl_2$ concentration screening of the origami folding. After folding, samples were loaded onto a 2% agarose gel (contains 0.5 mg mL^{-1} ethidium bromide and 10 mM $MgCl_2$) for electrophoresis (in 0.5X TBE supplemented with 10 mM $MgCl_2$, 90 volts for 2 hours). The gel was imaged under a UV channel.

6. Gel electrophoresis of origami before and after UV irradiation

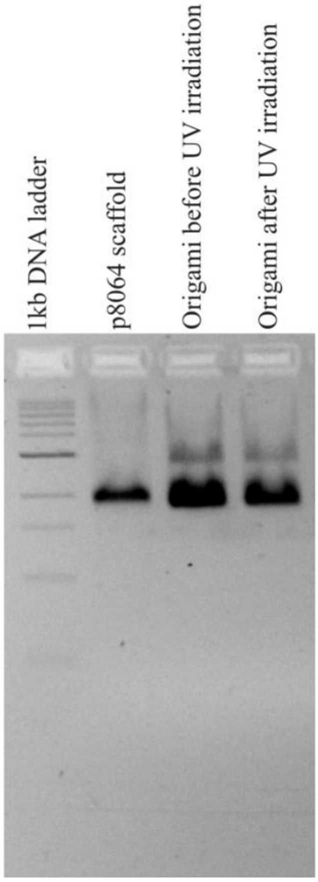

Supplementary Fig.6: Electrophoresis of the origami before and after experiencing UV irradiation. Samples were loaded onto a 2% agarose gel (contains 0.5 mg mL^{-1} ethidium bromide and 10 mM $MgCl_2$) for electrophoresis (in 0.5X TBE supplemented with 10 mM $MgCl_2$, 90 volts for 2 hours). The gel was imaged under a UV channel.

7. Negative-stain TEM image of the origami before UV irradiation in 5mM $MgCl_2$

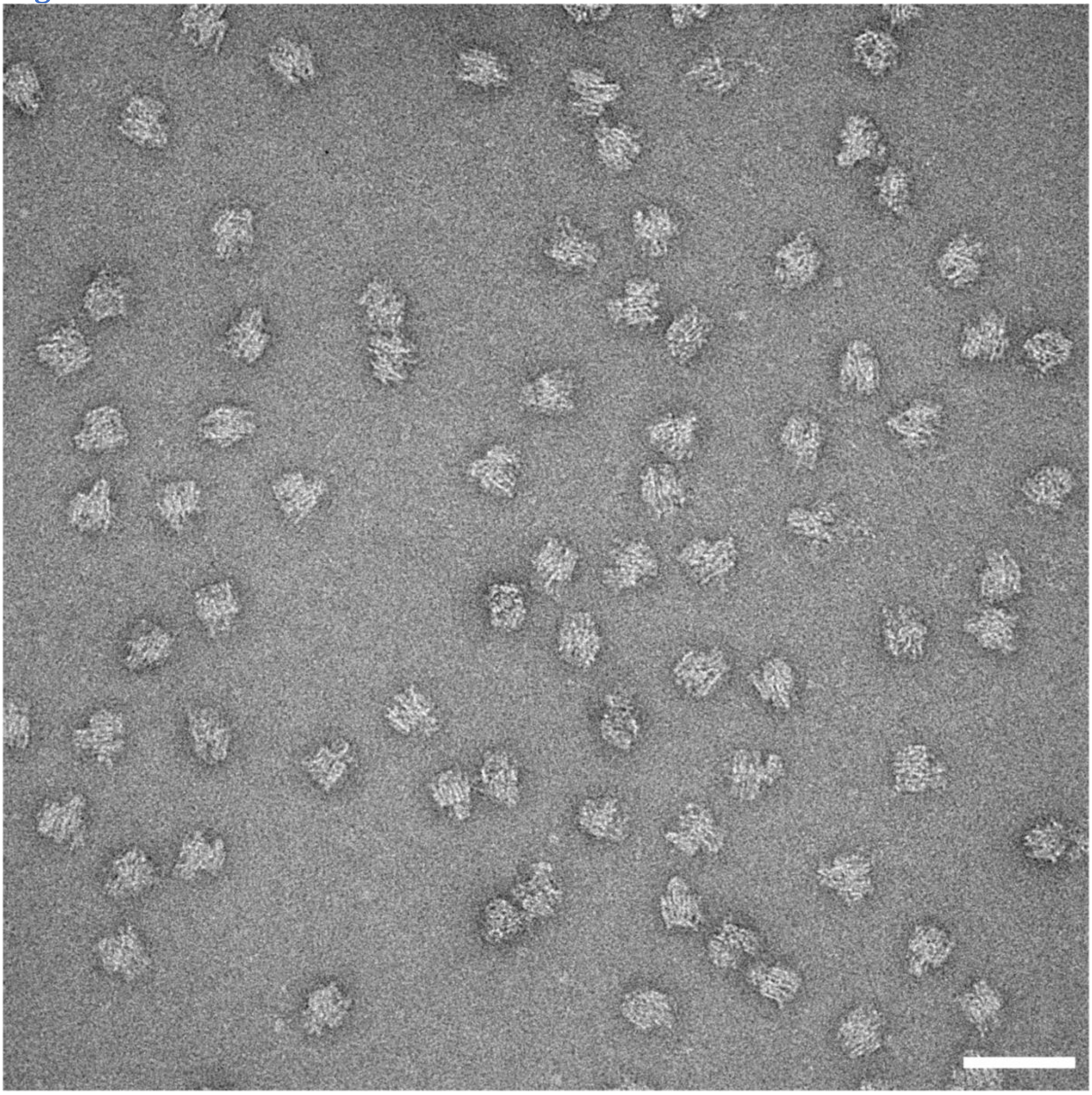

Supplementary Fig.7: Negative-stain TEM image of the origami (in the buffer contains 5 mM $MgCl_2$, 5 mM TRIS, and 1 mM EDTA) before UV irradiation. The scale bar stands for 100 nm.

8. Negative-stain TEM image of the origami before UV irradiation (buffer exchanged from 5 mM $MgCl_2$ into 1X PBS)

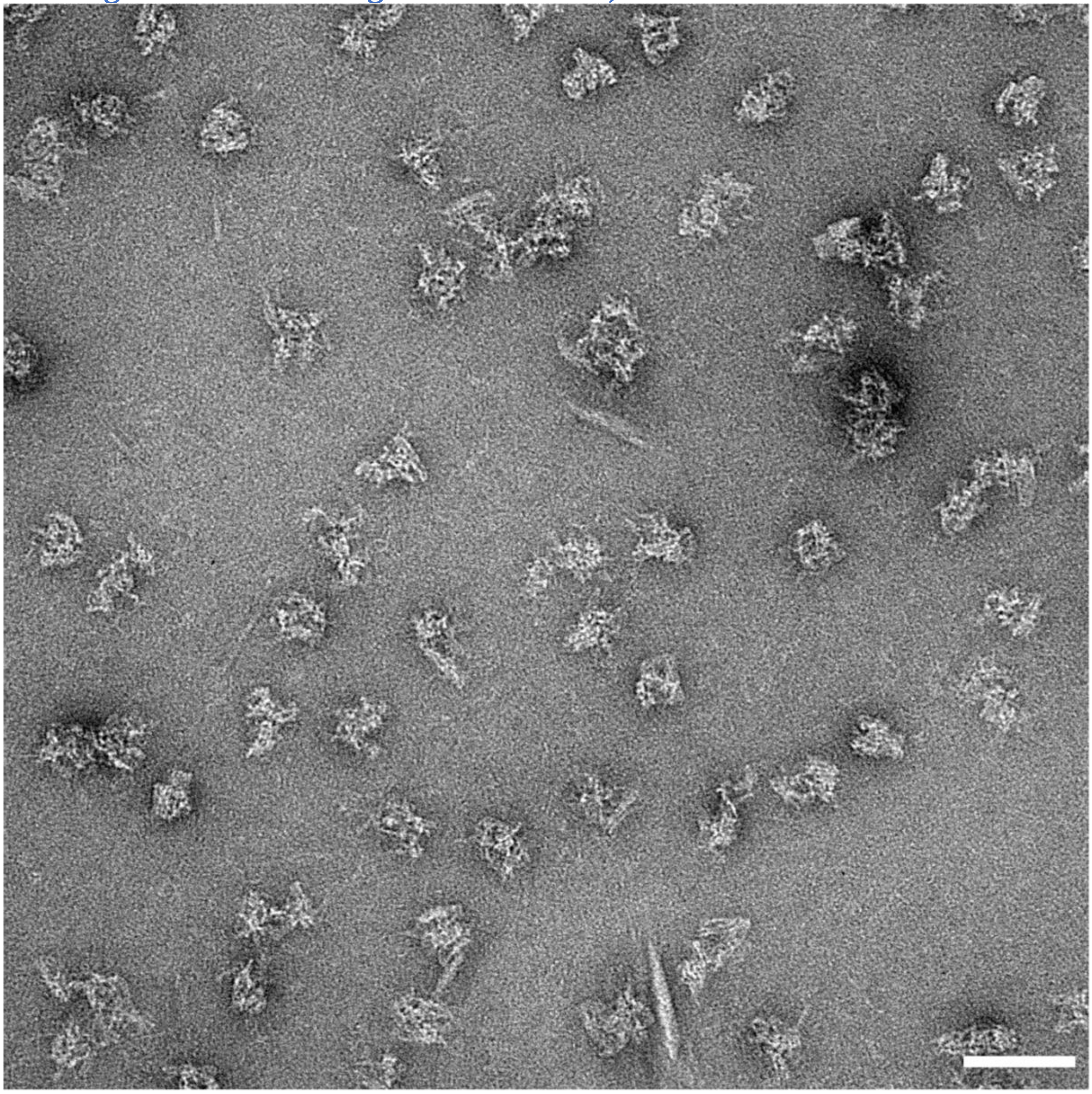

Supplementary Fig.8: Negative-stain TEM image of the origami (buffer exchanged from the buffer containing 5 mM MgCl2, 5 mM TRIS, and 1 mM EDTA to 1X PBS, then kept at room temperature overnight) before UV irradiation. The scale bar stands for 100 nm.

9. Negative-stain TEM image of UV-irradiated origami in 5 mM $MgCl_2$

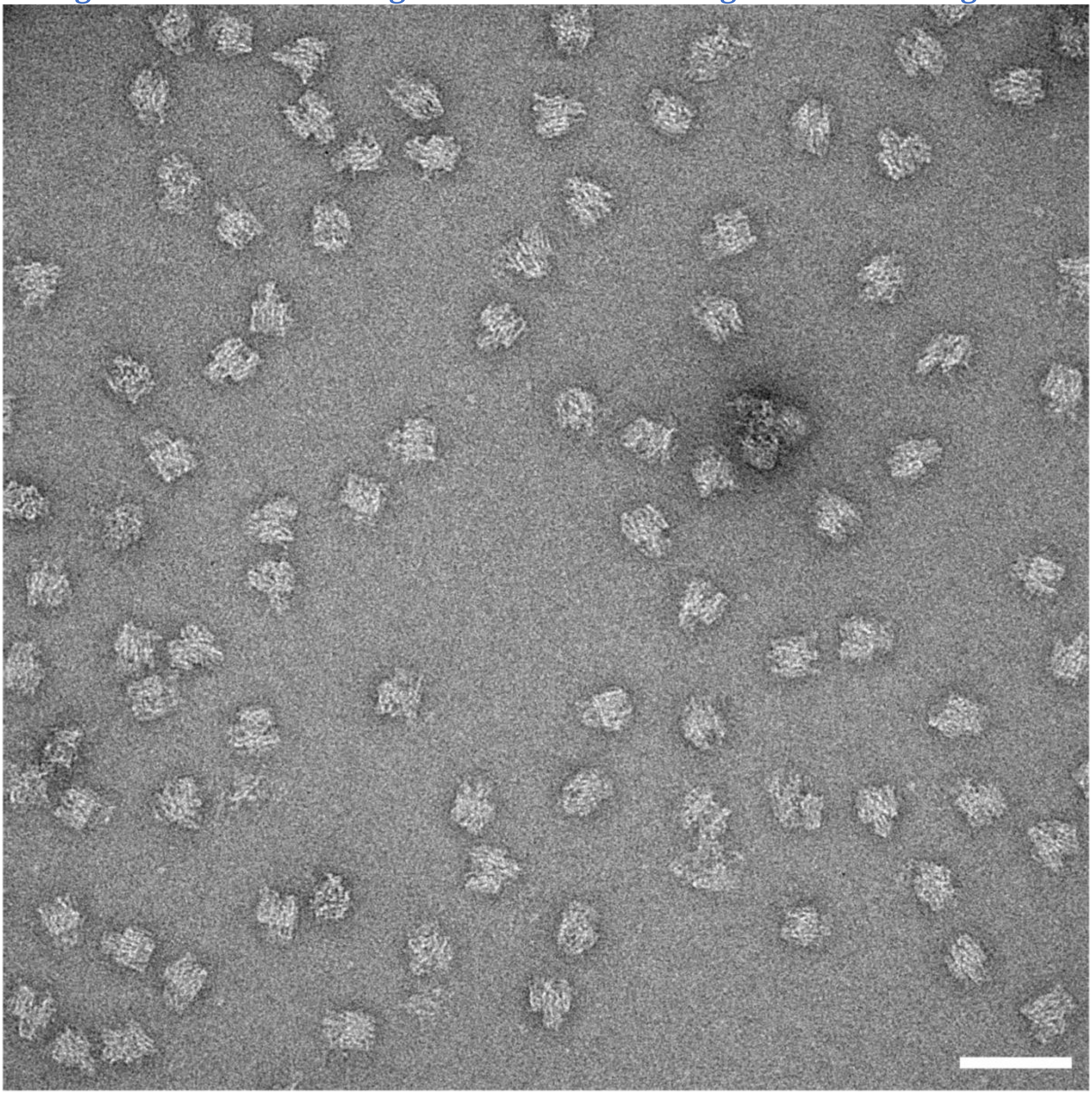

Supplementary Fig.9: Negative-stain TEM image of the origami (in the buffer contains 5 mM $MgCl_2$, 5 mM TRIS, and 1 mM EDTA) after UV irradiation. The scale bar stands for 100 nm.

10. Negative-stain TEM image of UV-irradiated origami in 1X PBS

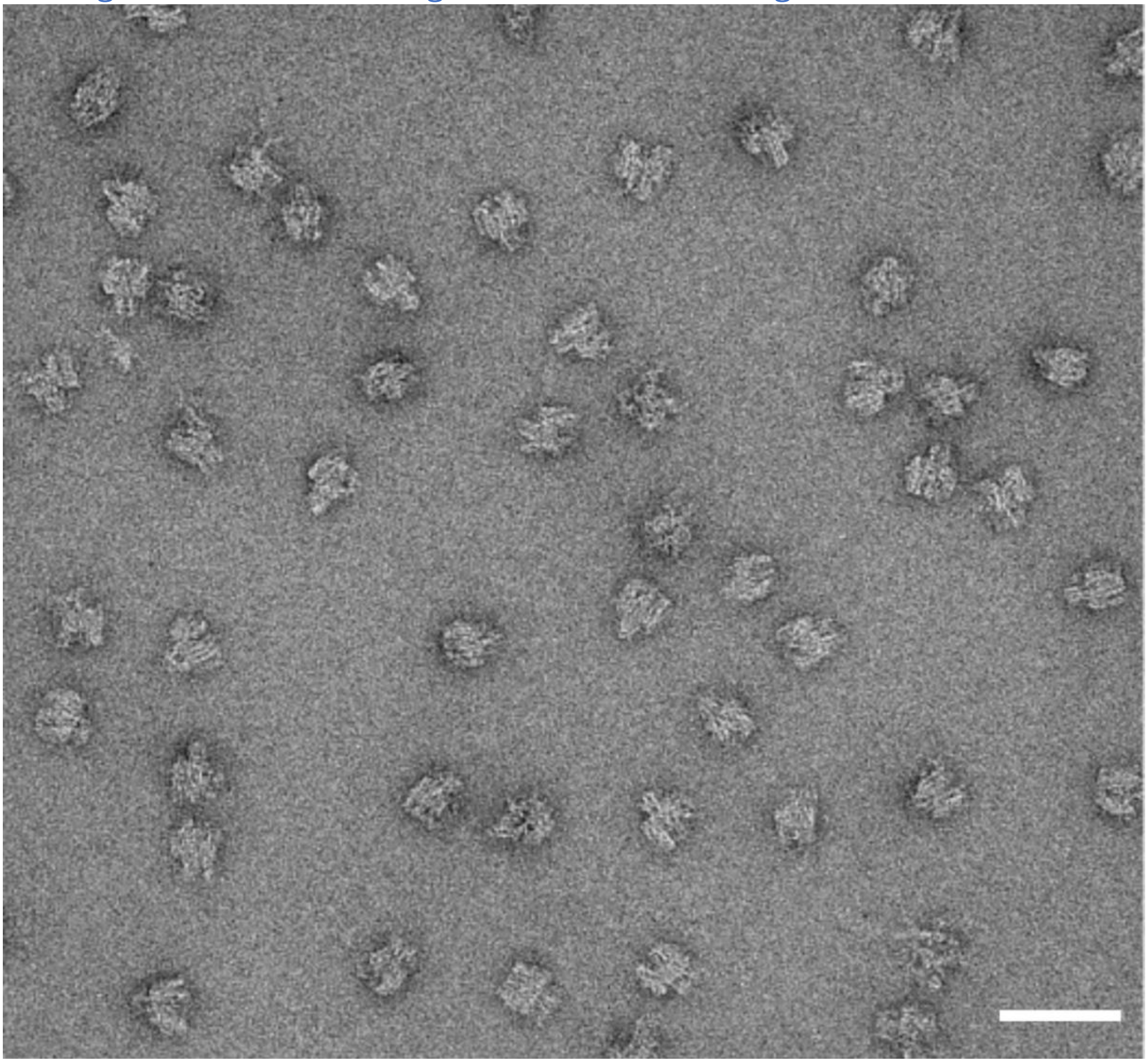

Supplementary Fig.10: Negative-stain TEM image of the origami (buffer exchanged from the buffer containing 5 mM $MgCl_2$, 5 mM TRIS, and 1 mM EDTA to 1X PBS, then kept at room temperature overnight) after UV irradiation. The scale bar stands for 100 nm.

11. Cryo-EM dataset pre-processing and processing workflow

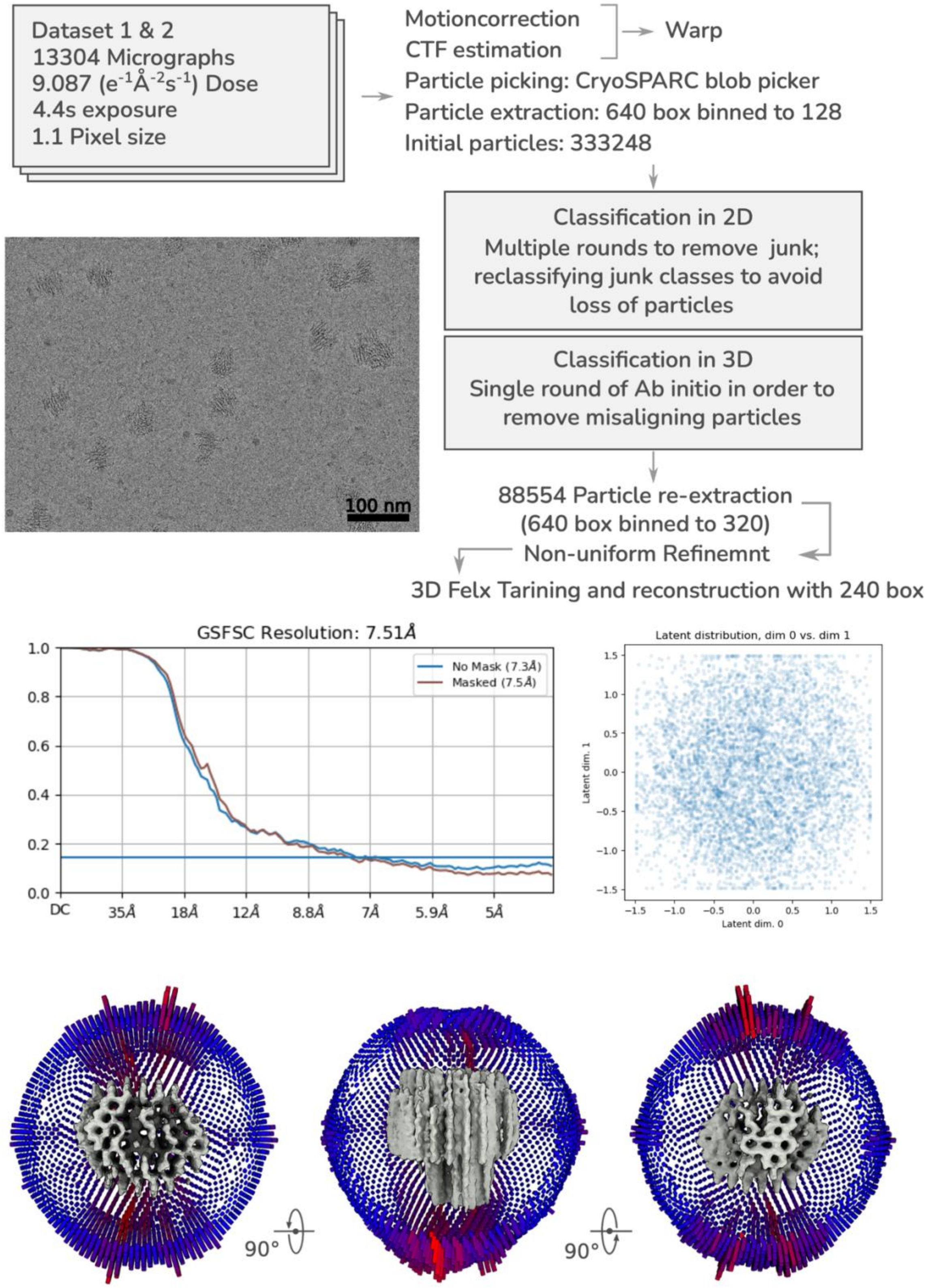

Supplementary Fig.11: Outline detailing the pre-processing and processing resulting in the consensus refinement and flexibility analysis. In the outline, a full representative micrograph can be seen; the consensus reconstruction obtained using 3D Flex tools, as well as, the view distribution showing the angular assignment of particles into the map; the FSC curves; and the latent distribution from the 3DFlex analysis showing the mode of movement.

12. Mini-Scaffold quantification of the UV-crosslinked origami

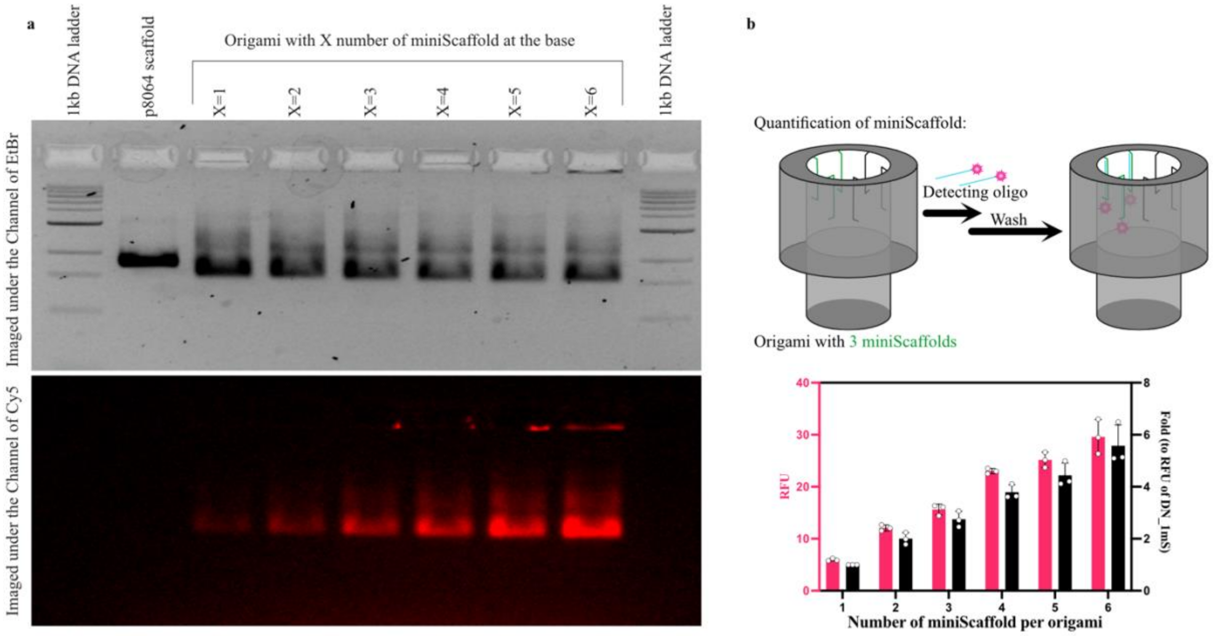

Supplementary Fig.12: Quantification of mini-scaffold in origami. Origami structures that contain 1, 2, 3, 4, 5, or 6 mini-Scaffold were prepared and purified. Then they were incubated with a Cy5-labeled oligo that detects the mini-Scaffold. After purification, the same amounts of origami samples were loaded onto a 2% agarose gel (contains 0.5 mg mL^{-1} ethidium bromide and 10 mM $MgCl_2$) for electrophoresis (in 0.5X TBE supplemented with 10 mM $MgCl_2$, 90 volts for 2 hours). The gel was imaged under a UV channel (a, top) and a fluorescent channel (a, bottom). The Cy5 signal of each origami sample was also quantitatively measured using a multimode microplate reader (b). n = 3 independent measurements. Data are presented as mean ± standard deviation.

13. FRET effects of the origami containing only one DNA triplex.

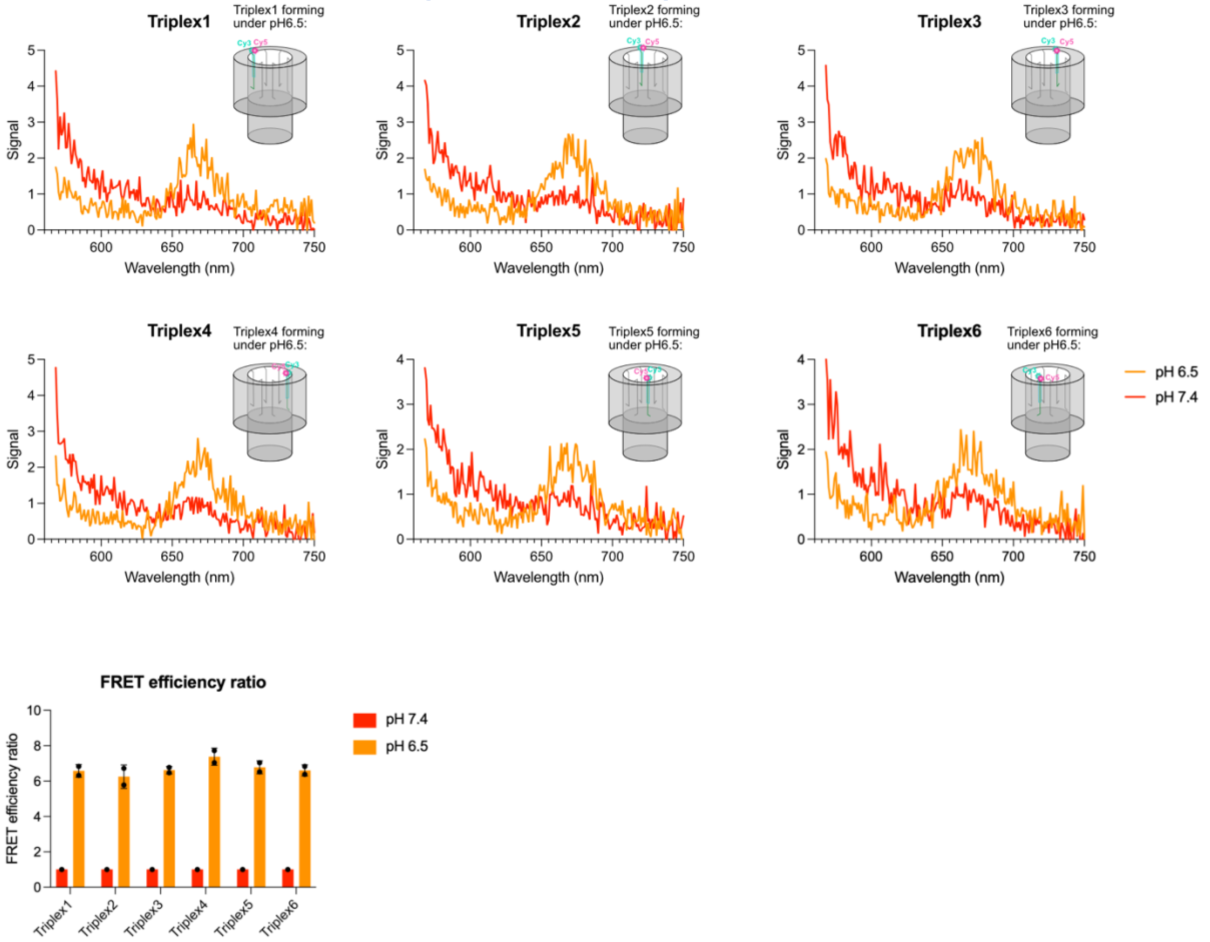

Supplementary Fig.13: FRET effects. With an excitation of 550 nm, the representative emission spectrum (from 568 nm to 750 nm) of the origami samples containing only 1 pH sensitive triplex switch of the hexagonal pattern under pH 7.4 or 6.5. The bar plot at the bottom of the figure shows the FRET efficiency ratio of each case (n = 2 independent measurements).

14. Conjugation of the peptide with DNA

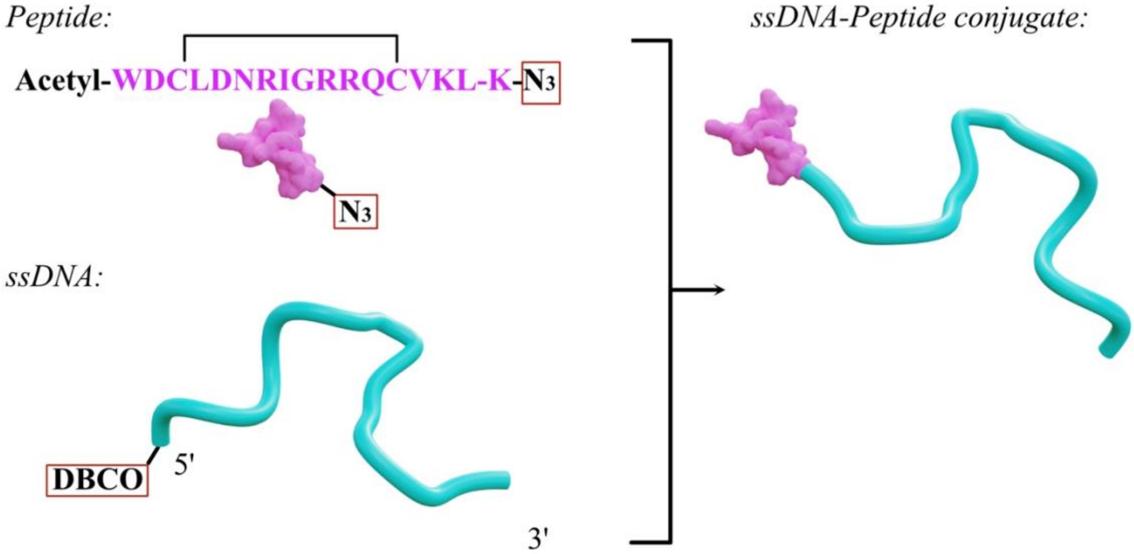

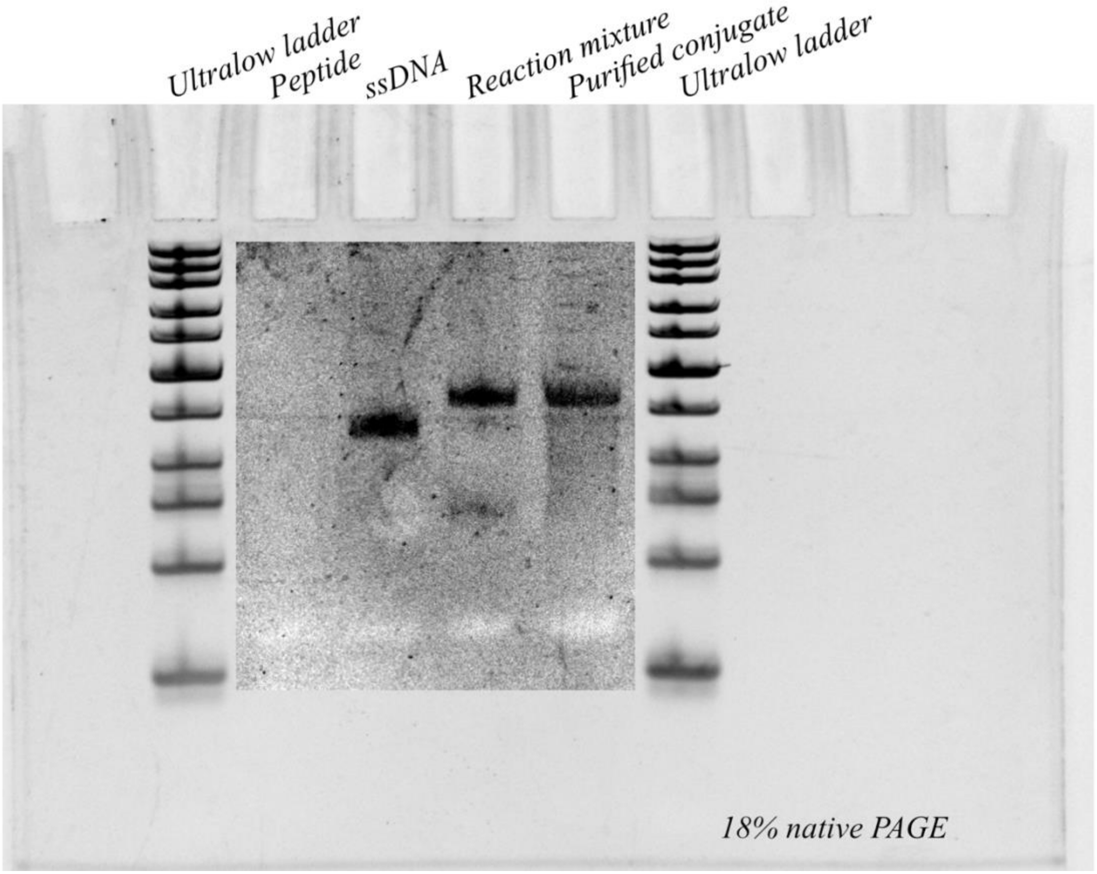

Supplementary Fig.14: Click chemistry-based conjugation of the N_3-modified cyclic peptide and DBCO-modified ssDNA. After the click reaction, the conjugate was purified via a proFIRE system (Dynamic Biosensors) and ran on a 18% native PAGE gel. The gel was stained with SYBR Gold before the imaging. The shift of the conjugate relative to the DNA indicates successful conjugation. To show the result clearly, the contrast of the central area of the gel is set to be higher than the rest of the image using ImageJ.

15. The affinity of the peptide-DNA conjugate to DR5 under different pH

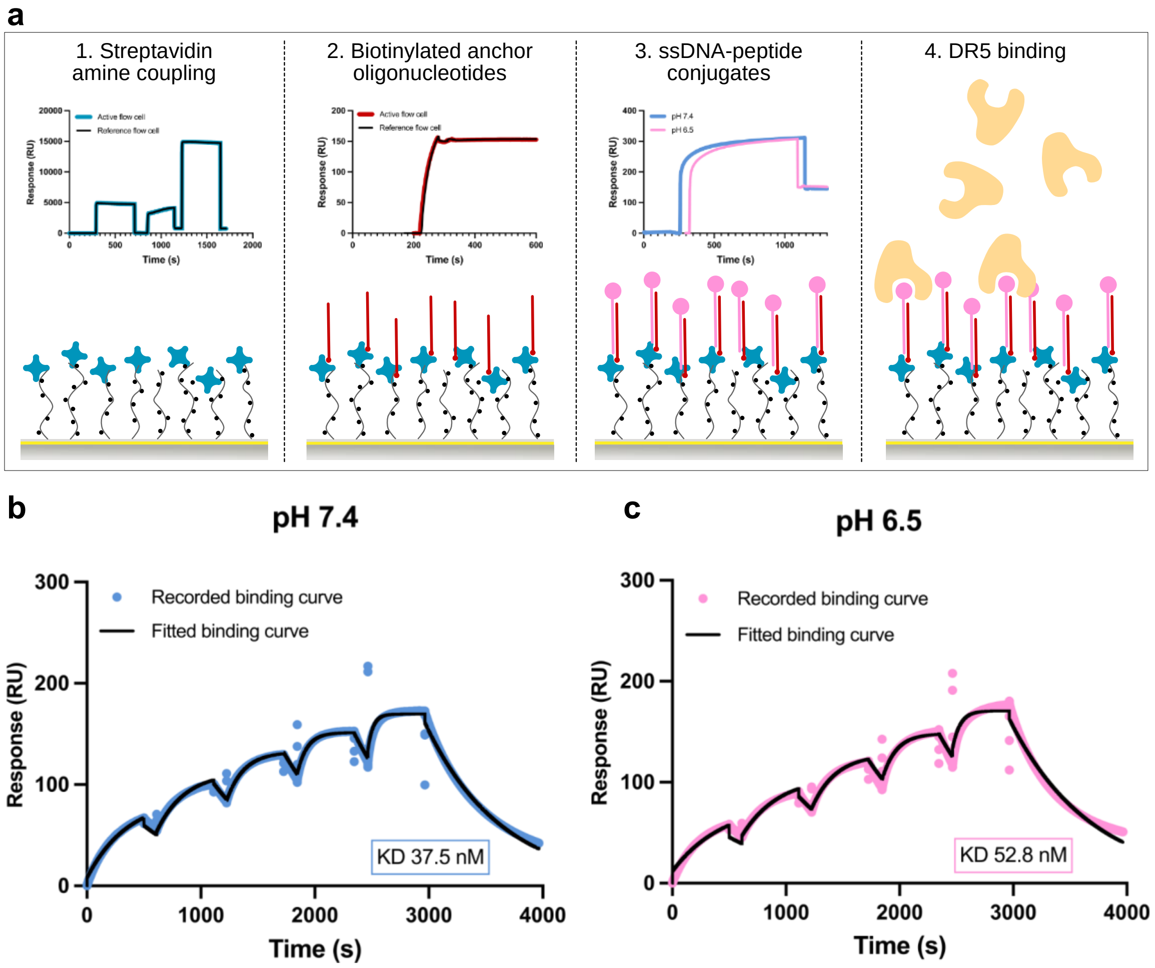

Supplementary Fig.15: Binding assay of ssDNA-peptide conjugate to the DR5 protein under different pH using SPR. (a) Schematic of the steps followed for the affinity measurements: 1. The CM3 chip was functionalized with streptavidin using amine-coupling in two flow cells, active (blue) and reference (black). 2. The biotinylated anchor oligonucleotides were immobilized in the two parallel flow cells, with sensograms shown for the active cell (red) and the reference cell (black). 2. The ssDNA-peptide conjugates were hybridized to the anchors via DNA complementarity in the active flow cell. The sensograms of ssDNA-peptide conjugate capture are shown for the runs at pH 7.4 (blue) and pH 6.5 (pink). 4. Finally, the DR5 protein was injected at five different concentrations. The association and dissociation curves of the different DR5 concentrations were recorded at pH 7.4 (b) and pH 6.5 (c), and analyzed with a 1:1 Langmuir model (fit in black). The dissociation constants KD, in colored boxes, are shown below the sensograms.

16. Peptide abundance assay on gel

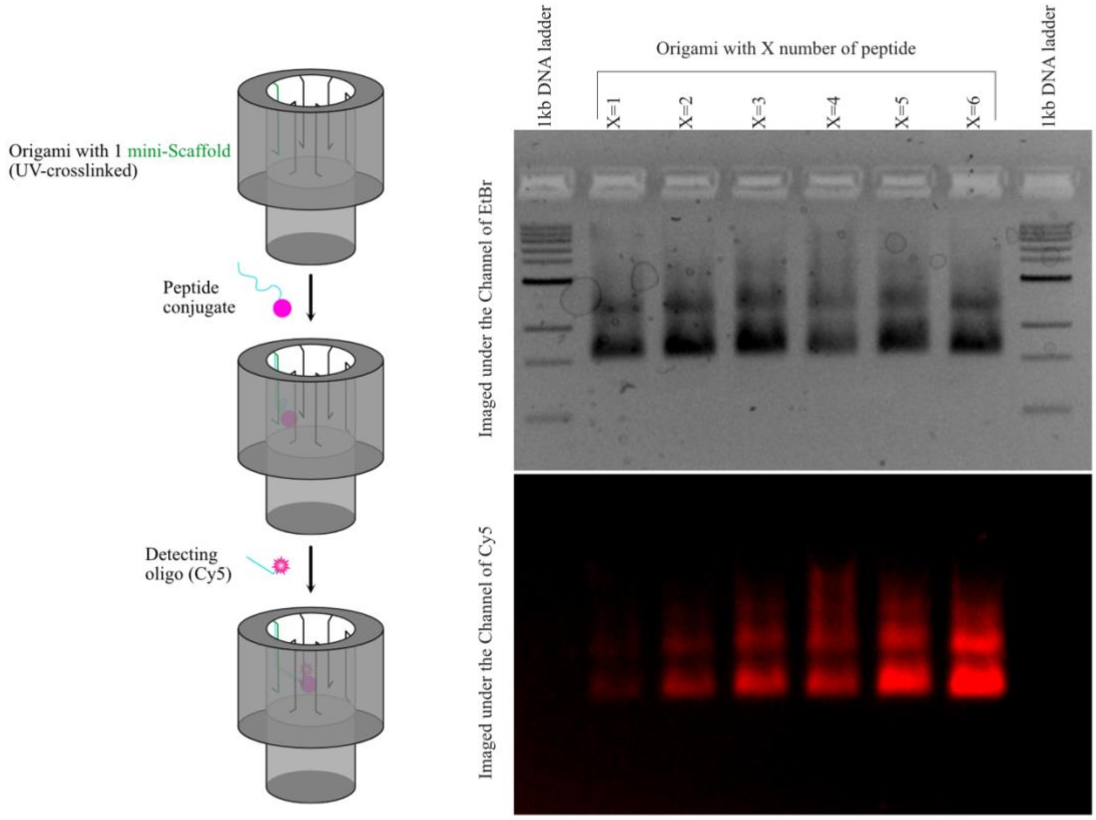

Supplementary Fig.16: Quantification of peptide in origami. Origami structures that contain 1, 2, 3, 4, 5, or 6 mini-scaffold were prepared and purified. Then they were incubated with the ssDNA-peptide conjugate that "dock" on the mini-Scaffolds. After purification, the same amounts of origami samples were further incubated with the Cy5-labeled oligo that targets the TFO of the ssDNA-peptide conjugate. After purification, the same amounts of origami samples were loaded onto a 2% agarose gel (contains 0.5 mg mL^{-1} ethidium bromide and 10 mM $MgCl_2$) for electrophoresis (in 0.5X TBE supplemented with 10 mM $MgCl_2$, 90 volts for 2 hours). The gel was imaged under a UV channel and a fluorescent channel.

17. The affinity of the origami displaying a peptide pattern to DR5

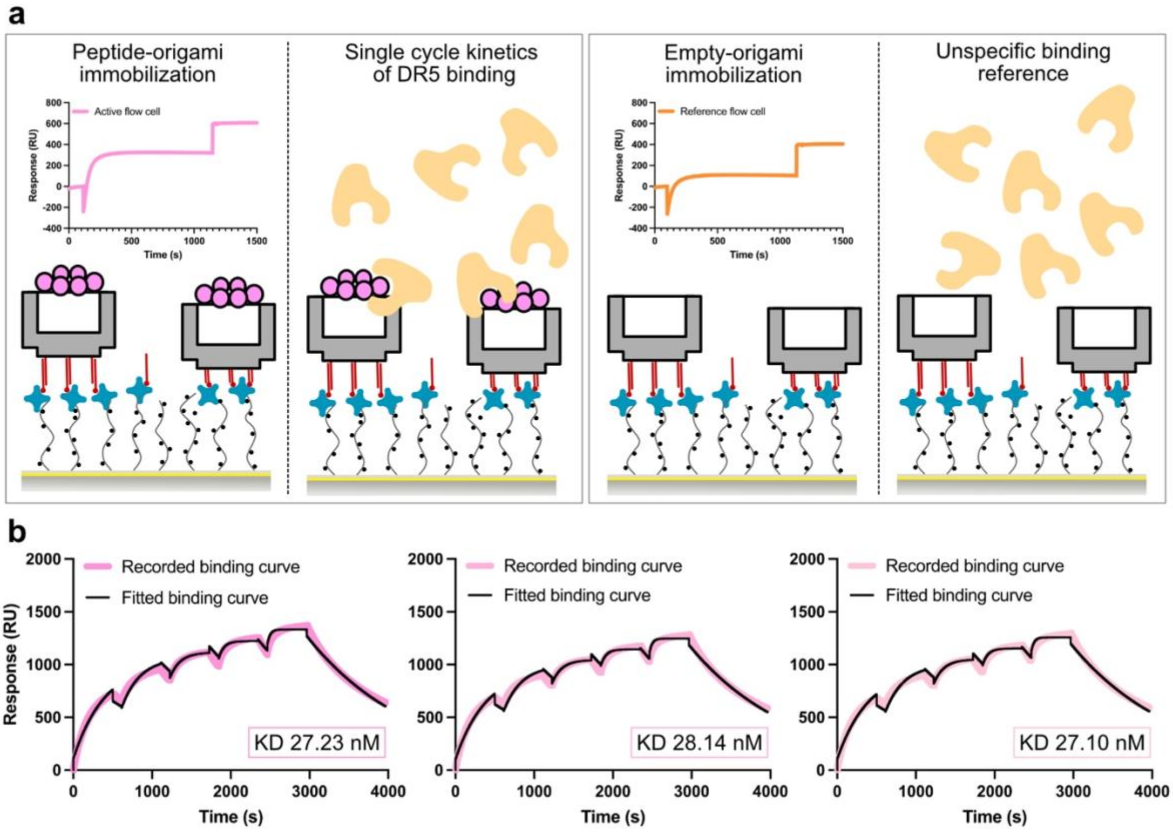

Supplementary Fig.17: SPR affinity measurement of the peptide-origami to the DR5 protein. (a) Representative sensograms of the peptide-origami (active flow cell) or empty origami (reference flow cell) immobilization prior to the single cycle kinetics assay with the DR5 protein. (b) The three replicates of the binding assay using a 2-fold dilution of the DR5 protein starting from 1032 nM. A 1:1 Langmuir model (fit in black) was used to analyze the data and the calculated dissociation constants KD for each run are shown in colored boxes below the sensograms. The average dissociation constant is 27.49 nM.

18. Cell interaction of the origami

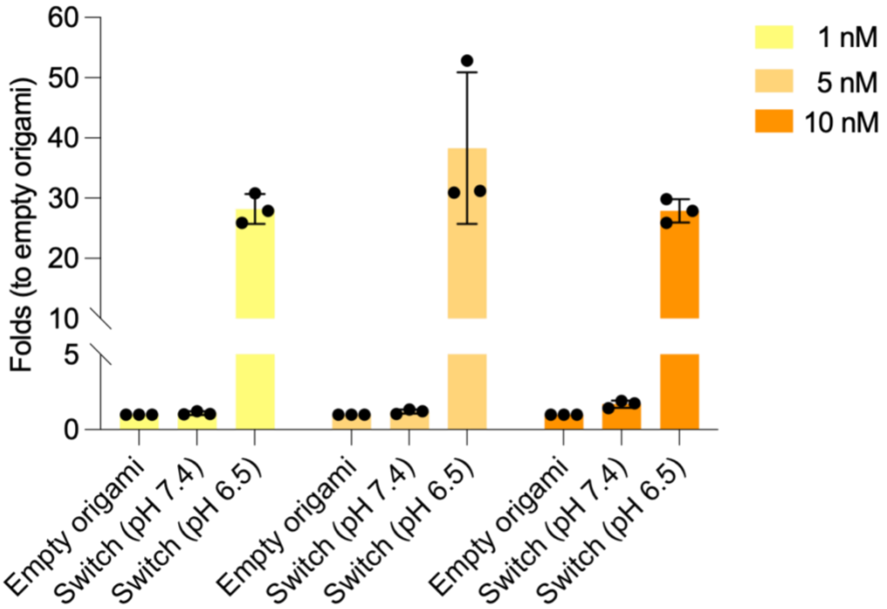

Supplementary Fig.18: Folds of origami associated with SK-BR-3 cells. This is measured using flow cytometry to measure the Cy5 fluorescent signal on cells. Before the measurement, cells were incubated with three different concentrations (1 nM, 5 nM, and 10 nM) of empty origami or origami switch (n = 3 independent measurements, data are presented as mean ± standard deviation).

19. Origami quantification per cell using repair qPCR

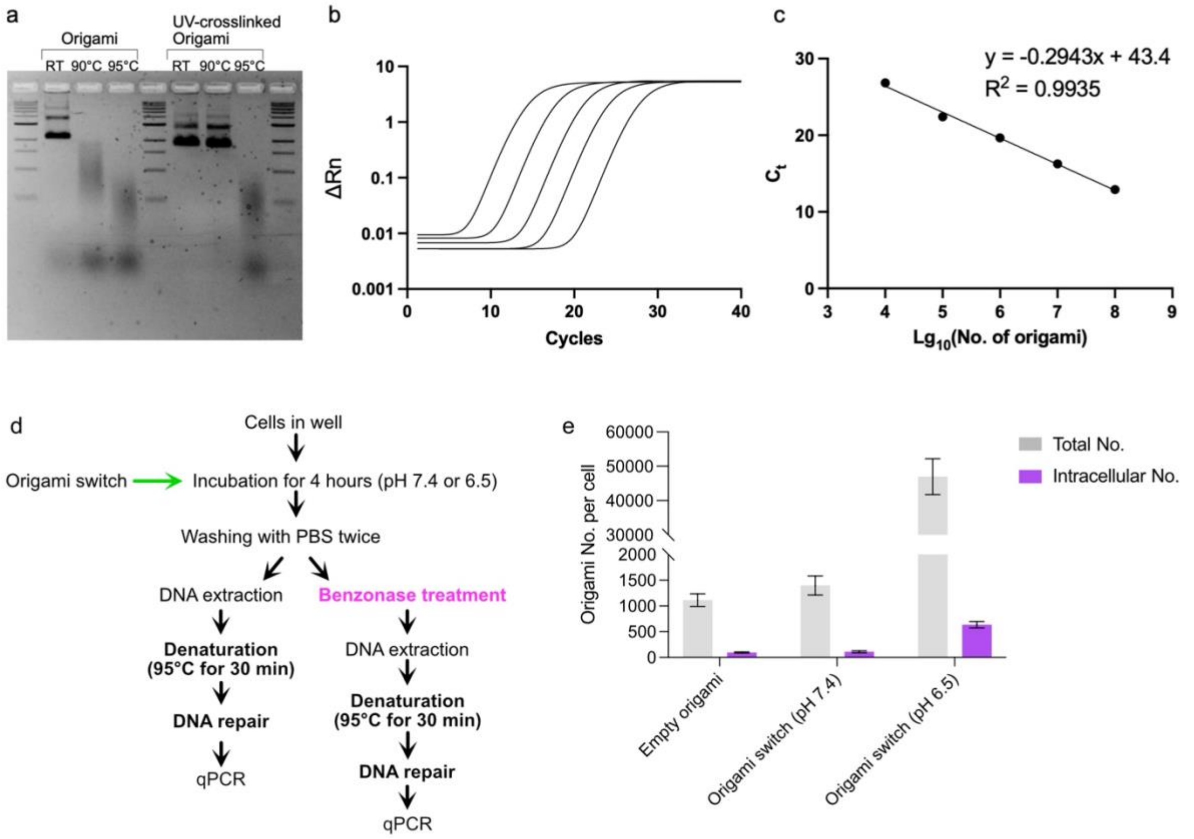

Supplementary Fig.19: Origami quantification on SK-BR-3 cells using repair qPCR. (a) 2% agarose gel electrophoresis to check the disassembly of UV-crosslinked origami after heat denaturation (90°C or 95°C) for 30 minutes. RT: room temperature. (b) Fluorescence plotted against number of PCR cycles for dilutions of UV-crosslinked DNA origami structures (Denatured under 95°C for 30 minutes, followed by repairing using the PreCR® Repair Mix) ranging from 104–108. (c) PCR Cycle thresholds plotted against the logarithm of initial number of origamis. R is the coefficient of determination (R squared). (d) Cellular experimental flows that quantify total origami amount on cells or intracellular origami amount. (e) Quantification of origami associated with cells under pH 7.4 and 6.5. Data are presented as mean ± standard deviation. (n = 2 independent measurements).

20. Tumor growth of the mice treated via intravenous injection

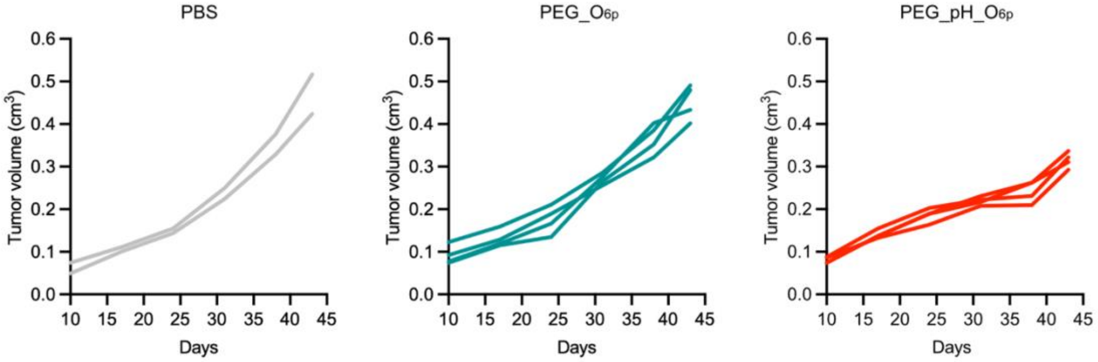

Supplementary Fig.20: Individual tumor growth curves from mice that received intravenous injections of PBS, PEG_mutated_O_{6p}, or PEG_pH_O_{6p}.

21. Tumor growth of the mice treated via intratumoral injection

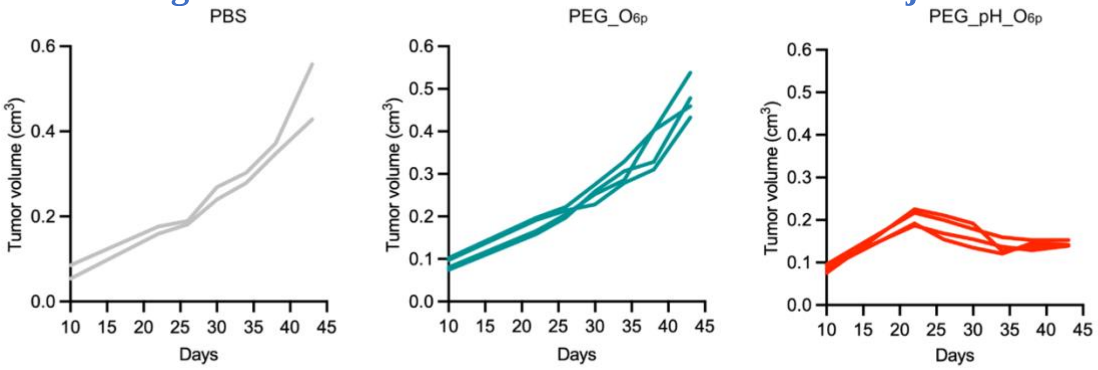

Supplementary Fig.21: Individual tumor growth curves from mice that received intratumoral injections of PBS, PEG_mutated_O_{6p}, or PEG_pH_O_{6p}.

22. Cleaved caspase-3 and 8 levels in the tumors receiving intratumoral injection

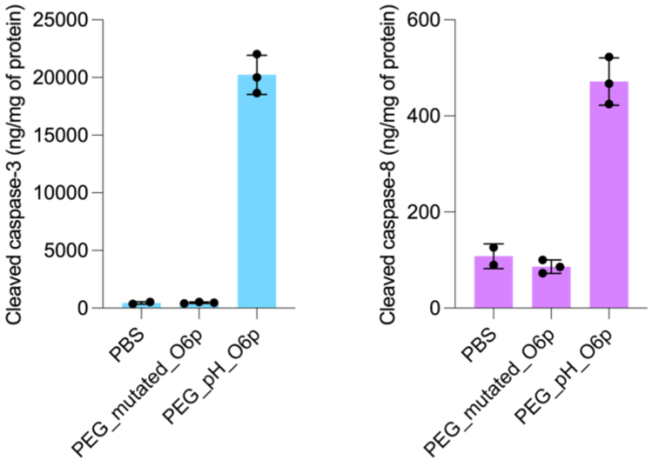

Supplementary Fig.22: The level of cleaved caspase-3 (left, using the kit: Human/Mouse Cleaved Caspase-3 (Asp175) DuoSet IC ELISA, R&D SYSTEMS, DYC835-2) and cleaved caspase-8 (right, using the kit: Human Cleaved CASP8 ELISA Kit, Biorbyt, orb564766) in tumors received intratumoral injections of PBS, PEG_mutated_O_{6p}, or PEG_pH_O_{6p}. Each dot represents a tumor tissue from a specific mouse. Data are presented as mean ± standard deviation. n = 2 for the PBS group; n = 3 for the PEG_mutated_O_{6p} group; n = 3 for the PEG_pH_O_{6p} group.

Uncropped gels showing in SI:

Supplementary Fig.5:

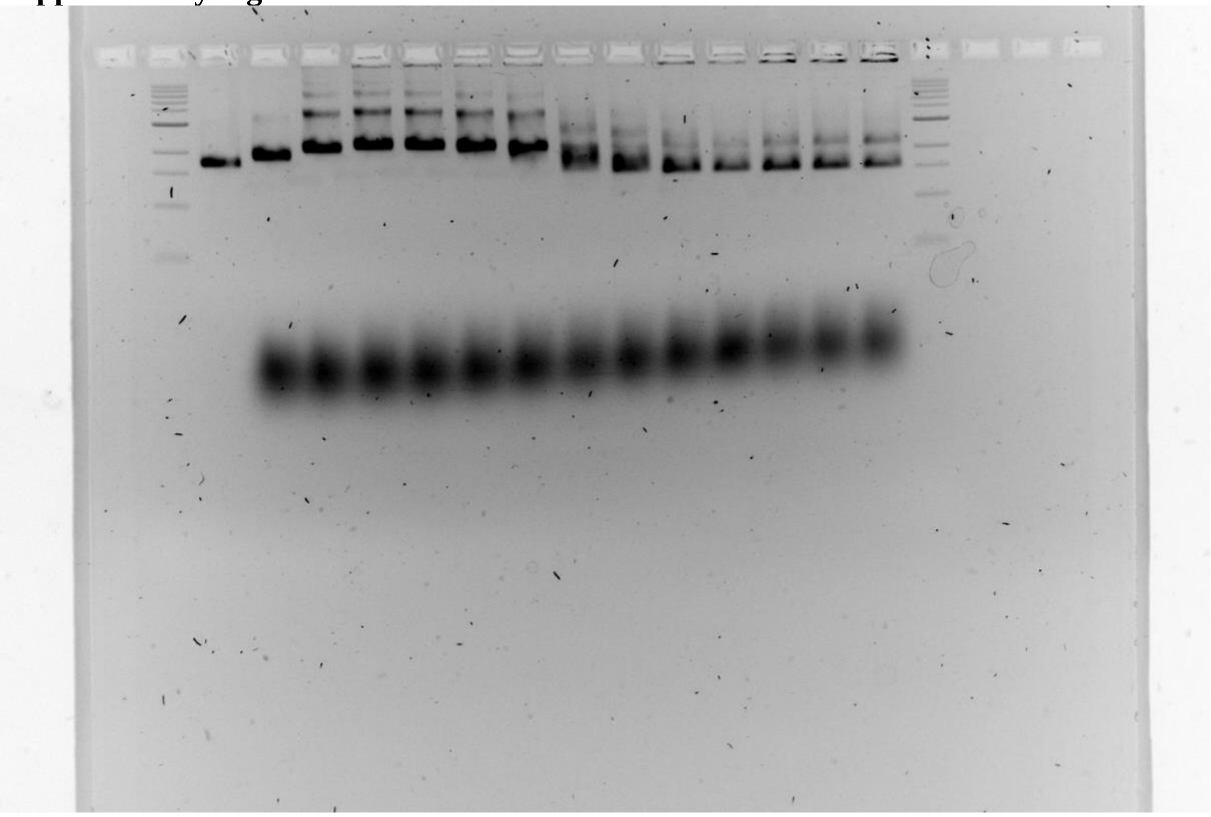

Supplementary Fig.6:

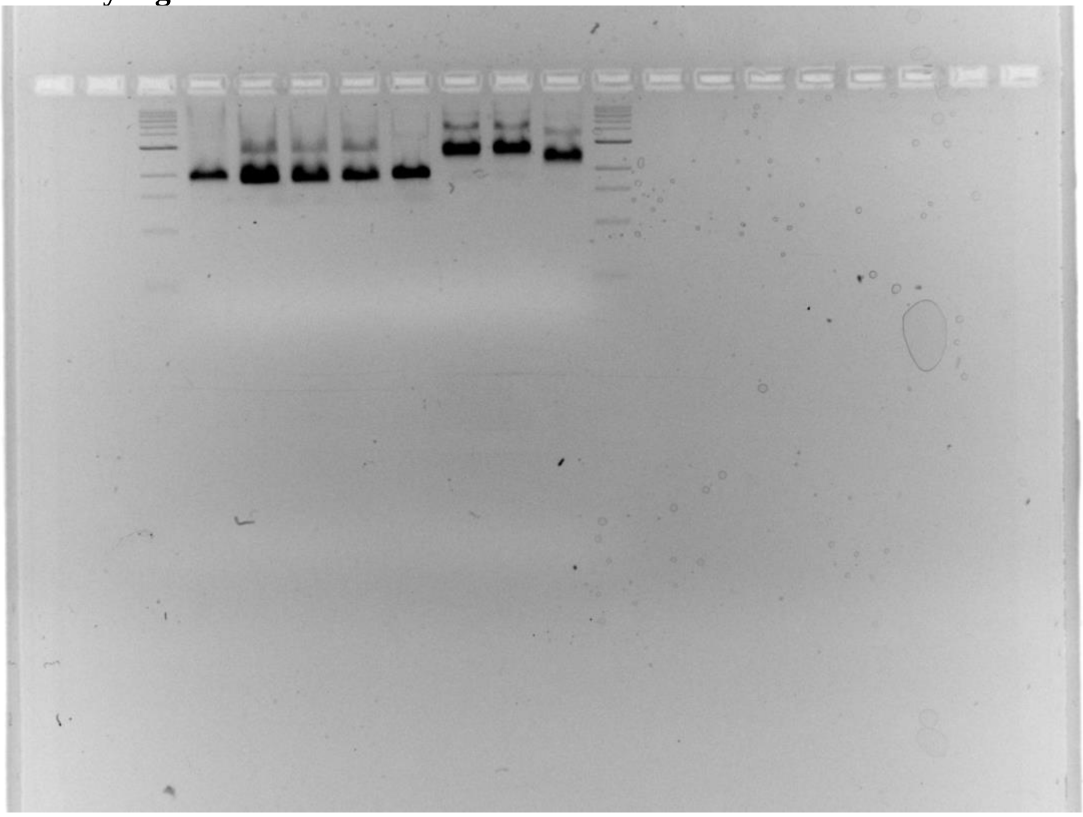

Supplementary Fig.12:

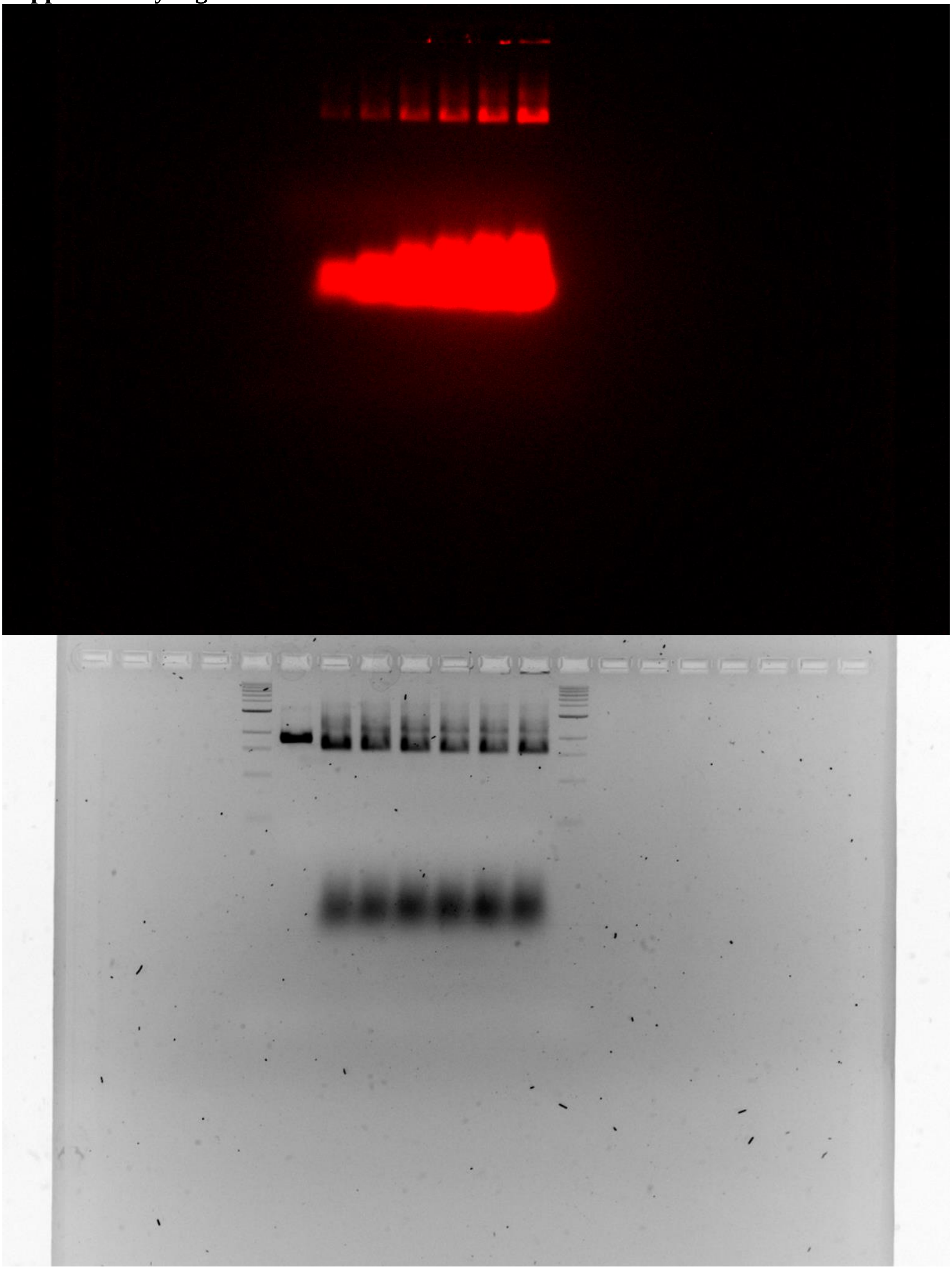

Supplementary Fig.14:

Supplementary Fig.16:

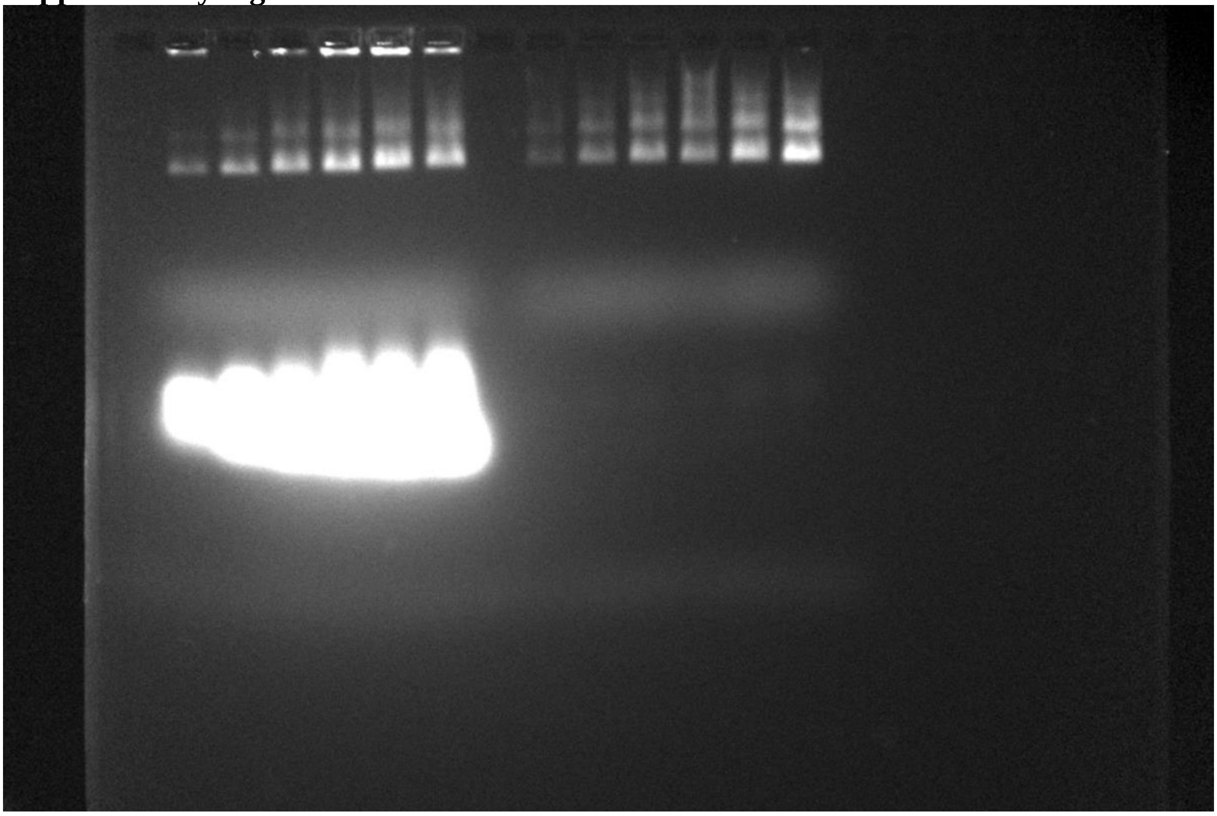

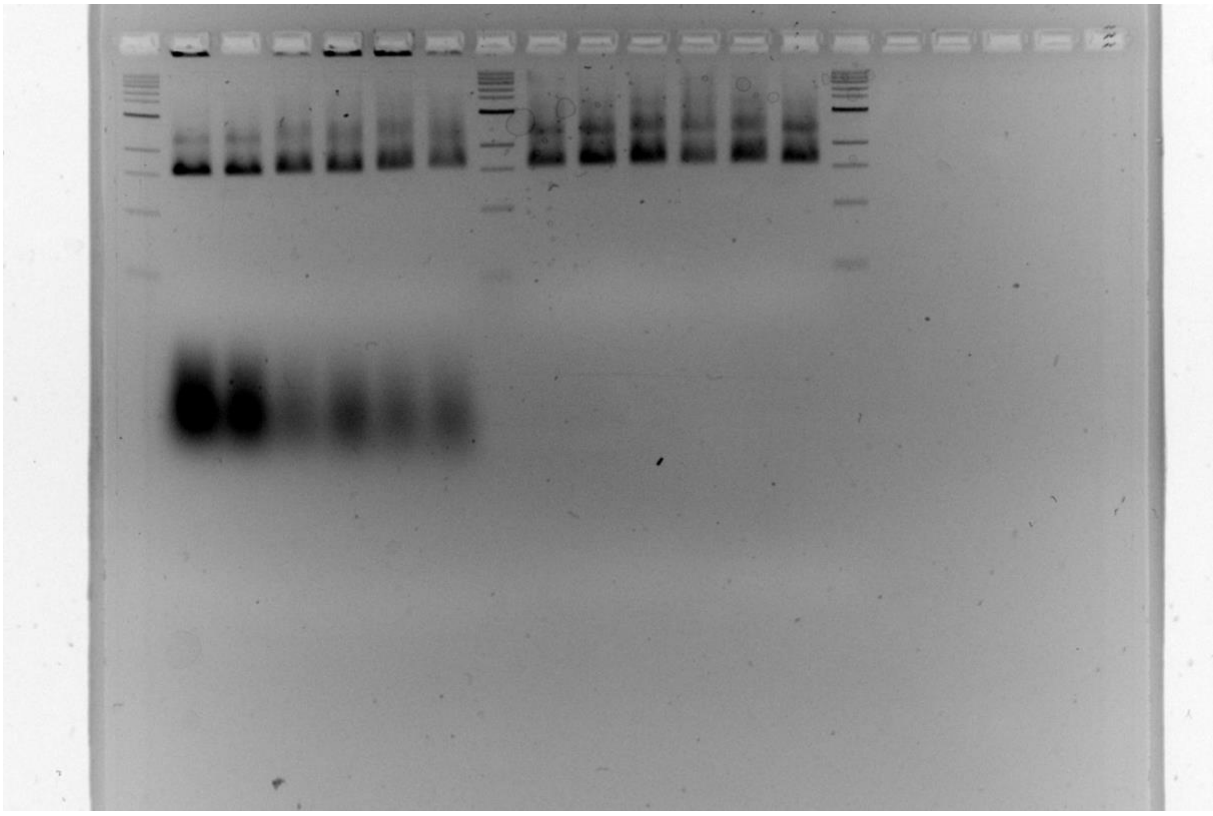

Supplementary Fig.19:

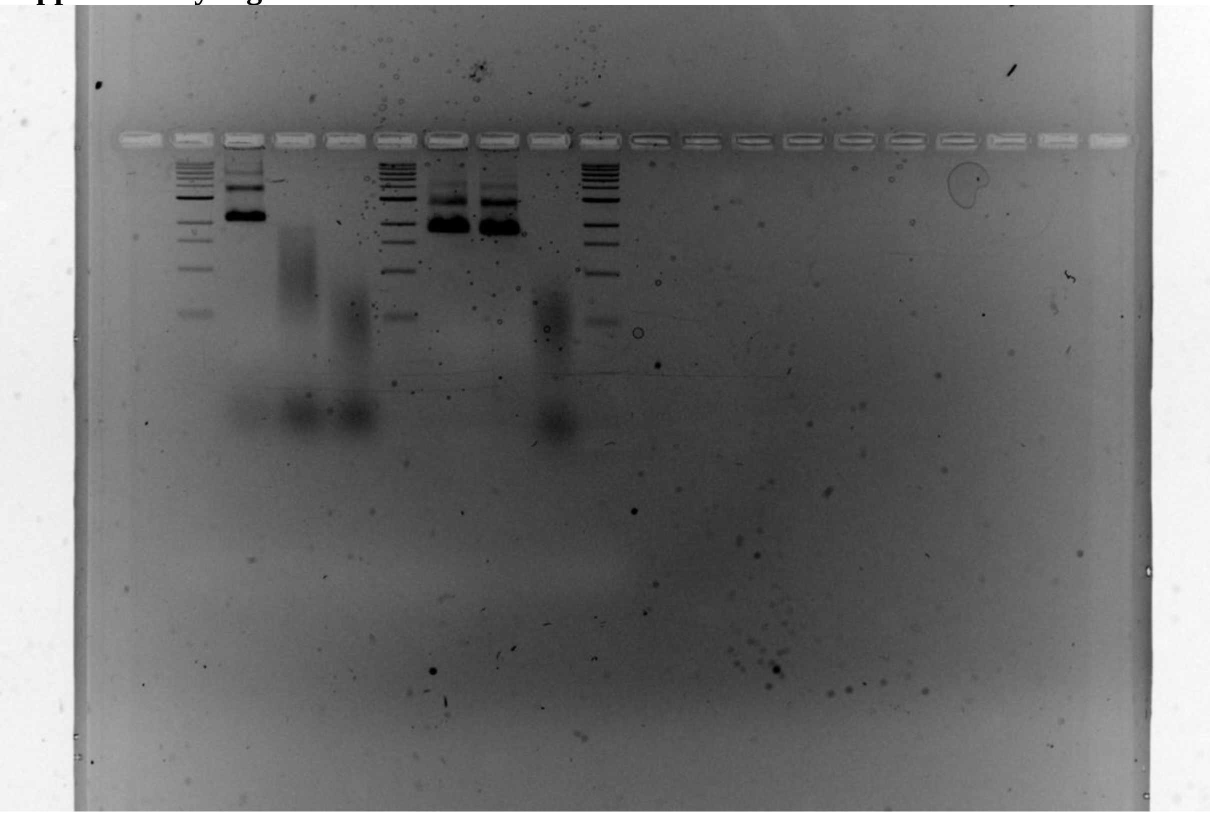

Supplement: Supplementary file 1 — Supplementary Figs. 1–22 and uncropped gel images. [file 41565_2024_1676_MOESM1_ESM.pdf]
