# Supplementary material for: A DNA robotic switch with regulated autonomous display of cytotoxic ligand nanopatterns
Source: Nat Nanotechnol. 2024 Jul 1;19(9):1366–74. doi: 10.1038/s41565-024-01676-4 (PMC11405282; doi:10.1038/s41565-024-01676-4)
Supplement: Supplementary file 2 — Reporting Summary [file 41565_2024_1676_MOESM2_ESM.pdf]

nature portfolio

Corresponding author(s): Björn Högberg

Last updated by author(s): Feb 1, 2024

Reporting Summary

Nature Portfolio wishes to improve the reproducibility of the work that we publish. This form provides structure for consistency and transparency in reporting. For further information on Nature Portfolio policies, see our Editorial Policies and the Editorial Policy Checklist.

Statistics

For all statistical analyses, confirm that the following items are present in the figure legend, table legend, main text, or Methods section.

n/a	Confirmed	
☐	☒	The exact sample size (n) for each experimental group/condition, given as a discrete number and unit of measurement
☐	☒	A statement on whether measurements were taken from distinct samples or whether the same sample was measured repeatedly
☐	☒	The statistical test(s) used AND whether they are one- or two-sided<br>*Only common tests should be described solely by name; describe more complex techniques in the Methods section.*
☒	☐	A description of all covariates tested
☐	☒	A description of any assumptions or corrections, such as tests of normality and adjustment for multiple comparisons
☐	☒	A full description of the statistical parameters including central tendency (e.g. means) or other basic estimates (e.g. regression coefficient) AND variation (e.g. standard deviation) or associated estimates of uncertainty (e.g. confidence intervals)
☐	☒	For null hypothesis testing, the test statistic (e.g. F, t, r) with confidence intervals, effect sizes, degrees of freedom and P value noted<br>*Give P values as exact values whenever suitable.*
☒	☐	For Bayesian analysis, information on the choice of priors and Markov chain Monte Carlo settings
☒	☐	For hierarchical and complex designs, identification of the appropriate level for tests and full reporting of outcomes
☒	☐	Estimates of effect sizes (e.g. Cohen's d, Pearson's r), indicating how they were calculated

Our web collection on statistics for biologists contains articles on many of the points above.

Software and code

Policy information about availability of computer code

Data collection	Peptide-DNA conjugates were purified by using a proFIRE system (Dynamic Biosensors).<br>For SPR experiments, data was acquired using Biacore T200 System Control software v.2.01.<br>Gels were imaged under a GE LAS 4000 imager.<br>Negative-stain TEM images were collected in a 120 kV LaB6 microscope (Talos 120C G2 with Ceta-D detector).<br>Vitrobot Mk4 (FEI) was used to prepare cryo specimens.<br>Cryo-EM data were collected with EPU using a Krios G3i TEM operated at 300kV. Images were acquired in 81 kx nanoprobe EFTEM SA mode with a slit width of 10eV using a K3 Bioquantum.<br>Confocal images were collected using LSM700 (Zeiss).<br>Flow cytometry data was collected using the Cytek Aurora Flow Cytometer.<br>The luminescence was recorded on a multimode microplate reader (Varioskan LUX).<br>Biodistribution of the origami,were imaged using a Perkin Elmer in vivo imaging system (IVIS).
Data analysis	GraphPad Prism v9.4.0, v9.3.1, oxDNA coarse-grained modelling (https://oxdna.org/), oxView tool (https://oxdna.org/), tacoxDNA (http://tacoxdna.sissa.it/), FlowJo v10.7.1 (BD Biosciences), ImageJ/Fiji v1.53.

For manuscripts utilizing custom algorithms or software that are central to the research but not yet described in published literature, software must be made available to editors and reviewers. We strongly encourage code deposition in a community repository (e.g. GitHub). See the Nature Portfolio guidelines for submitting code & software for further information.

Data

Policy information about availability of data

All manuscripts must include a data availability statement. This statement should provide the following information, where applicable:

- Accession codes, unique identifiers, or web links for publicly available datasets
- A description of any restrictions on data availability
- For clinical datasets or third party data, please ensure that the statement adheres to our policy

The DNA origami design is available in the supplement as well as deposited to nanobase.org with accession number #233, https://nanobase.org/structure/233. The electron density maps of the UV-crosslinked robotic switch are available in the Electron Microscopy Data Bank (EMDB) as entry EMD-19129. Source data are provided with this paper.

Research involving human participants, their data, or biological material

Policy information about studies with human participants or human data. See also policy information about sex, gender (identity/presentation), and sexual orientation and race, ethnicity and racism.

Reporting on sex and gender	n/a
Reporting on race, ethnicity, or other socially relevant groupings	n/a
Population characteristics	n/a
Recruitment	n/a
Ethics oversight	n/a

Note that full information on the approval of the study protocol must also be provided in the manuscript.

Field-specific reporting

Please select the one below that is the best fit for your research. If you are not sure, read the appropriate sections before making your selection.

☒ Life sciences ☐ Behavioural & social sciences ☐ Ecological, evolutionary & environmental sciences

For a reference copy of the document with all sections, see nature.com/documents/nr-reporting-summary-flat.pdf

Life sciences study design

All studies must disclose on these points even when the disclosure is negative.

Sample size	No statistical methods were used to pre-determine sample sizes. All sample sizes are listed in each figure legend.
Data exclusions	No data were excluded from analysis
Replication	Reproducibility was ensured by sampling from multiple biological replicates. The exact number of independent biological experiments or number of lavae used for each measurement is mentioned in the figure legends. No results are included that were not observed in multiple experiments. All attempts at replication were successful.
Randomization	For experiments with cell cultures, cells were cultured in wells, and then randomly allocated to treatment and control groups. For SPR experiments randomization was not applicable, since they cannot be allocated into different experimental groups. Mice bearing xenografts were randomly assigned to treatment groups.
Blinding	Investigators were not blind to subject groups because knowledge of experimental conditions was required during data collection and evaluation.

Reporting for specific materials, systems and methods

We require information from authors about some types of materials, experimental systems and methods used in many studies. Here, indicate whether each material, system or method listed is relevant to your study. If you are not sure if a list item applies to your research, read the appropriate section before selecting a response.

Materials & experimental systems

n/a	Involved in the study	
☐	☒	Antibodies
☐	☒	Eukaryotic cell lines
☒	☐	Palaeontology and archaeology
☐	☒	Animals and other organisms
☒	☐	Clinical data
☒	☐	Dual use research of concern
☒	☐	Plants

Methods

n/a	Involved in the study	
☒	☐	ChIP-seq
☐	☒	Flow cytometry
☒	☐	MRI-based neuroimaging

Antibodies

Antibodies used	Antibodies (Supplier, Catalog No), concentration<br>human TRAIL R2/TNFRSF10B antibody (R&D systems, MBA6311), 66 nM<br>Goat Anti-Mouse IgG H&L (Alexa Fluor® 488) (Abcam, ab150113), 2 µg/ml
Validation	https://www.rndsystems.com/products/human-trail-r2-tnfrsf10b-antibody-71908_mab6311<br>https://www.abcam.com/products/secondary-antibodies/goat-mouse-igg-hl-alexa-fluor-488-ab150113.html

Eukaryotic cell lines

Policy information about cell lines and Sex and Gender in Research

Cell line source(s)	Cell lines were purchased from ATCC:<br>SK-BR-3: https://www.atcc.org/products/htb-30<br>Jurkat T: https://www.atcc.org/products/tib-152<br>HEK293: https://www.atcc.org/products/crl-1573<br>HBEC-5i: https://www.atcc.org/products/crl-3245
Authentication	Cell lines were authenticated by providers. We further authenticated this cell line by testing its ability to differentiate to adipocytes by evaluating their morphology, accumulation of lipids, and by qPCR analysis of fat-selective genes using species specific primers.
Mycoplasma contamination	Cell line was tested negative for mycoplasma contamination using LookOut Mycoplasma qPCR detection kit (Sigma)
Commonly misidentified lines<br>(See ICLAC register)	No commonly misidentified cell lines were used

Animals and other research organisms

Policy information about studies involving animals; ARRIVE guidelines recommended for reporting animal research, and Sex and Gender in Research

Laboratory animals	Female BALB/c Nude CByJ.Cg-Foxn1nu/J mice at the age of 29 days were ordered from Charles River and bred at the animal facility Comparative Medicine Biomedicum (KMB), Solna Campus, Karolinska Institutet.
Wild animals	n/a
Reporting on sex	Female mice were used in this study because breast cancer model was established.
Field-collected samples	n/a
Ethics oversight	All animal handling and experimental procedures were carried out according to local ethics guidelines and approved by Stockholm's Animal Experimentation Ethics Committee (Stockholms djurförsöksetiska nämnd, Dnr 16041-2019).

Note that full information on the approval of the study protocol must also be provided in the manuscript.

Plants

Seed stocks	n/a
Novel plant genotypes	n/a
Authentication	n/a

Flow Cytometry

Plots

Confirm that:

☒ The axis labels state the marker and fluorochrome used (e.g. CD4-FITC).

☒ The axis scales are clearly visible. Include numbers along axes only for bottom left plot of group (a 'group' is an analysis of identical markers).

☒ All plots are contour plots with outliers or pseudocolor plots.

☒ A numerical value for number of cells or percentage (with statistics) is provided.

Methodology

Sample preparation	For the quantitative assay of origami binding to cells：1 × 107 SK-BR-3 cells per well were cultured in 6-well plates overnight. The medium of each well was replaced by 2 mL of fresh medium with pH 7.4 or 6.5 containing 5 nM of Cy5-labeled origami structures for 4-hour treatments in a humidified incubator containing 5% CO2 at 37 °C. Cells were collected and washed twice with cold 1 × PBS, followed by the measurement with the Flow Cytometer. For the cell apoptosis assay：with 6-well plates, 1 × 107 SK-BR-3 cells were seeded in each well for culture overnight. The medium was removed, and 2 mL of fresh medium with pH 7.4 or 6.5 containing 5 nM origami was added to the cells for 24-hour treatments. All cells (including detached cells in the medium) were collected and resuspended in cold 1 × PBS. Cells were then stained with annexin V-FITC and PI, sequentially, according to the protocol of a commercial apoptosis assay kit (Thermo Fisher SCIENTIFIC, V13242). Cells were analyzed using the Cytek Aurora Flow Cytometer.
Instrument	Cytek Aurora Flow Cytometer
Software	FlowJo 10.7.1 Software (BD Biosciences)
Cell population abundance	The cells under different treating conditions showed significantly different populations in live cell, early apoptotic cell, late apoptotic cell and necrotic cell, which were expected to see. The abundances were high enough.
Gating strategy	The cells without treatments were used for gating based on SSC-A (y axis) and FSC-A (x axis). During analysis, different cell populations were selected and plotted onto an AnnexinV PE against a propidium iodide dot plot to access the levels of apoptosis.

☒ Tick this box to confirm that a figure exemplifying the gating strategy is provided in the Supplementary Information.
